# Biomimetic GBM-targeted drug delivery system boosting ferroptosis for immunotherapy of orthotopic drug-resistant GBM

**DOI:** 10.1186/s12951-022-01360-6

**Published:** 2022-03-27

**Authors:** Bao Liu, Qifeng Ji, Ying Cheng, Miao Liu, Bangle Zhang, Qibing Mei, Daozhou Liu, Siyuan Zhou

**Affiliations:** 1 Department of Pharmaceutics, School of Pharmacy, Air Force Medical University, Changle West Road 169, Xi’an, 710032 Shaanxi China; 2 Key Laboratory of Gastrointestinal Pharmacology of Chinese Materia Medica of the State Administration of Traditional Chinese Medicine, Department of Pharmacology, School of Pharmacy, Air Force Medical University, Changle West Road 169, Xi’an, 710032 Shaanxi China

**Keywords:** Drug-resistant glioblastoma, siPD-L1, Ferroptosis, Immunotherapy, Glioblastoma, Microglia

## Abstract

**Background:**

Clinical studies have shown that the efficacy of programmed cell death receptor-1/programmed cell death ligand-1 (PD-1/PD-L1) inhibitors on glioblastoma (GBM) is much lower than what is expected because of the low immunogenicity of GBM. Ferroptosis of cancer cells can induce the maturation of dendritic cells (DC cells) and increase the activity of T cell. The activated T cells release IFN-γ, which subsequently induces the ferroptosis of cancer cells. Thus, the aim of this paper is to set up a new GBM-targeted drug delivery system (Fe_3_O_4_-siPD-L1@M_-BV2_) to boost ferroptosis for immunotherapy of drug-resistant GBM.

**Results:**

Fe_3_O_4_-siPD-L1@M_-BV2_ significantly increased the accumulation of siPD-L1 and Fe^2+^ in orthotopic drug-resistant GBM tissue in mice. Fe_3_O_4_-siPD-L1@M_-BV2_ markedly decreased the protein expression of PD-L1 and increased the ratio between effector T cells and regulatory T cells in orthotopic drug-resistant GBM tissue. Moreover, Fe_3_O_4_-siPD-L1@M_-BV2_ induced ferroptosis of GBM cells and maturation of DC cell, and it also increased the ratio between M1-type microglia and M2-type microglia in orthotopic drug-resistant GBM tissue. Finally, the growth of orthotopic drug-resistant GBM in mice was significantly inhibited by Fe_3_O_4_-siPD-L1@M_-BV2_.

**Conclusion:**

The mutual cascade amplification effect between ferroptosis and immune reactivation induced by Fe_3_O_4_-siPD-L1@M_-BV2_ significantly inhibited the growth of orthotopic drug-resistant GBM and prolonged the survival time of orthotopic drug-resistant GBM mice.

**Graphical Abstract:**

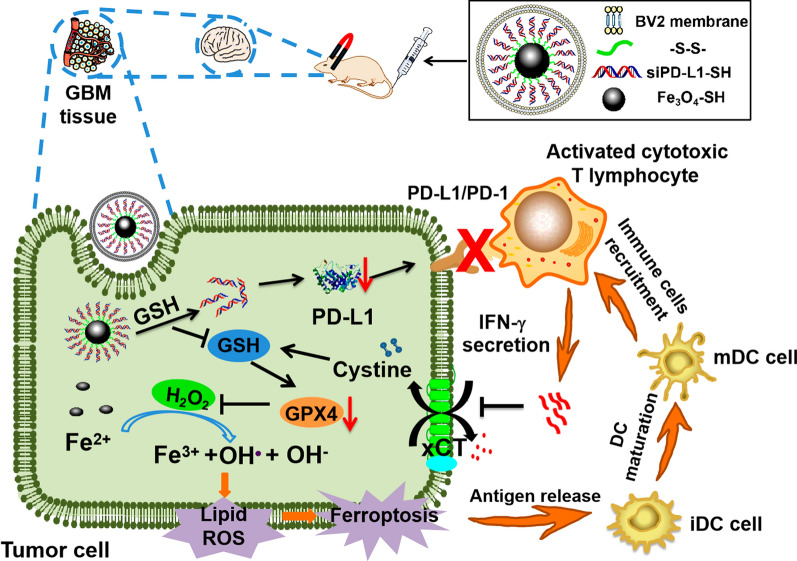

**Supplementary Information:**

The online version contains supplementary material available at 10.1186/s12951-022-01360-6.

## Background

Glioblastoma (GBM) is an aggressive intracranial malignant tumor with high mortality and morbidity, accounting for 80% of malignant tumors in central nervous system (CNS). The overall median survival for GBM patients is only about 15 months [1, 2]. At present, surgical resection followed by radiotherapy and temozolomide (TMZ) chemotherapy is considered to be the basic treatment for patients with newly diagnosed GBM [3–5]. However, it is frustrating that long-term use of TMZ in GBM patients inevitably leads to the overexpression of O^6^-methylguanine DNA methyltransferase (MGMT) in GBM cells, which results in the resistance of GBM cells to TMZ. Subsequently, the efficacy of TMZ is significantly reduced or even lost [6, 7]. Therefore, it is an urgent need to find new treatment methods for TMZ-resistant GBM.

Immunotherapy, a very promising cancer treatment method, inhibits tumor growth and metastasis by inducing systemic and sustained immune response [8]. However, the efficacy of immunotherapy on GBM is much lower than what is expected. This is resulted from the following reasons. Firstly, as compared with other cancer such as non-small cell lung cancer, GBM in most cases shows a lower tumor mutational burden [9], resulting in lower immunogenicity of GBM cells and less recruitment of effector T cells (T_eff_ cell) in GBM tissue [10]. Secondly, GBM cells usually recruit regulatory T cell (T_reg_ cell) into GBM tissue by secreting chemokines such as colony stimulating factor 1 (CSF1), C-X-C Motif Chemokine Ligand 12 (CXCL12), C-X-C Motif Chemokine Ligand 1 (CXCL1) and granulocyte–macrophage colony stimulating factor (GM-CSF) [11]. T_reg_ cell inhibits the function of T_eff_ cell, subsequently reducing the generation of interleukin-2 (IL-2) and interferon-γ (IFN-γ) [12, 13]. Finally, GBM cells are able to polarize anti-tumor M1 type microglia/macrophage into the immunosuppressive M2 type microglia/macrophage by secreting immunomodulatory cytokines [14, 15]. M2 type microglia/macrophage also inhibits the function of T_eff_ cell and promotes the progression of GBM by secreting cytokines such as interleukin-6 (IL-6), interleukin-10 (IL-10) and C–C Motif Chemokine Ligand 2 (CCL2) [16].

Ferroptosis is a form of iron-dependent cell death. The essences of ferroptosis are the over-load of Fe^2+^, depletion of glutathione (GSH) and the decrease of glutathione peroxidase (GPX4) [17, 18]. Lipid oxides cannot be metabolized through GPX4. Subsequently, a large number of hydroxyl radicals are produced through Fenton reaction, leading to lipid peroxidation in cancer cells. This finally results in cancer cell death [19, 20]. Many studies have shown that ferroptosis also leads to the maturation of DC cells in cancer tissue in vivo, and the matured DC cells present antigen to T lymphocytes to activated T_eff_ [21, 22]. Moreover, PD-1/PD-L1 inhibitor is able to activate T_eff_ cell to secrete IFN-γ [23]. IFN-γ secreted by activated T_eff_ cell inhibits the cysteine transporter (xCT) and subsequently prevents the cysteine from being taken up by cancer cells, resulting in the reduction of GSH synthesis in cancer cells. Then, ferroptosis is significantly enhanced in turn [24–26]. In theory, ferroptosis inducer and PD-1/PD-L1 inhibitor can mutually enhance each efficacy when they are simultaneously used to treat GBM.

siRNA shows high specificity and low toxicity in cancer treatment [27]. It has potential application value to interfere PD-L1 protein synthesis in drug-resistant GBM cells by using siPD-L1. However, lack of suitable siPD-L1 delivery vector and easy degradation of siPD-L1 in blood circulation are the main obstacles that limit the application of siPD-L1 in the treatment of GBM [28, 29]. As compared with other carriers, Fe_3_O_4_ nanoparticle is a promising siPD-L1 carrier [30]. Firstly, Fe_3_O_4_ nanoparticle displays good biocompatibility and biodegradability, and it is easily available. Fe_3_O_4_ nanoparticle has been approved for clinic use by the Food and Drug Administration (FDA) [31]. Secondly, Fe_3_O_4_ nanoparticle significantly increases the intracellular iron content especially Fe^2+^ [32, 33], which provides sufficient substrate for ferroptosis in drug-resistant GBM cells. Last, Fe_3_O_4_ nanoparticle shows super paramagnetism, which allows it to be directed delivery by an external magnetic field [34]. However, Fe_3_O_4_ nanoparticle is difficult to cross blood–brain barrier (BBB) [35, 36]. Recent studies have shown that GBM tissue can recruit microglia by secreting chemokines such as C-X3-C motif chemokine ligand 1 (CX3CL1) and CSF-1 [37–39]. In theory, microglia membrane coated Fe_3_O_4_ nanoparticle can be recruited to drug-resistant GBM.

**Scheme 1.**
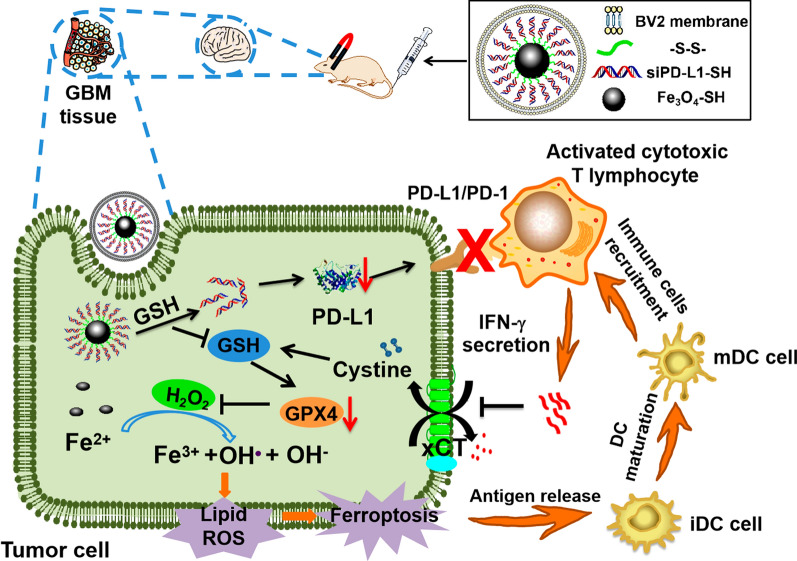
Reciprocal cascade amplification between ferroptosis and immunotherapy of Fe_3_O_4_-siPD-L1@M_-BV2_

In this study, disulfide bonds were used to connect thiolated siPD-L1 and thiolated Fe_3_O_4_ nanoparticles to increase the stability of siPD-L1 in blood circulation. Fe_3_O_4_ nanoparticles connected with siPD-L1 (Fe_3_O_4_-siPD-L1) are further coated with microglial membrane (M_-BV2_) to form a biomimetic brain-targeted nanoparticle Fe_3_O_4_-siPD-L1@M_-BV2_. After Fe_3_O_4_-siPD-L1@M_-BV2_ was taken up by orthotopic drug-resistant GBM cells, the disulfide bond between Fe_3_O_4_ nanoparticles and siPD-L1 was broken by intracellular GSH, releasing siPD-L1 and inhibiting the protein expression of PD-L1 in orthotopic drug-resistant GBM cells [40]. Subsequently, T_eff_ cell was activated to enhance the killing effect on drug-resistant GBM cell [41]. At the same time, Fe_3_O_4_-siPD-L1@M_-BV2_ facilitated the ferroptosis of drug-resistant GBM cells, which further activated T_eff_ cell by improving the maturation of DC cells. Moreover, activated T_eff_ cell enhanced the ferroptosis of drug-resistant GBM cells in turn by secreting IFN-γ. Finally, there forms a cascade amplification effect between ferroptosis and immune activation in orthotopic drug-resistant GBM tissue (Scheme 1).

## Materials and methods

### Materials

Thiolated Fe_3_O_4_ nanoparticle was bought from Ruixibio (Xi’an, China). Thiolated siPD-L1 (5’-GAAGGGAAAUGCUGCCCUUTT-3’, 5’-AAGGGCAUUUCCCUUCTT-3’) and cyanine5 labeled siPD-L1 were obtained from GenePharma (Shanghai, China). PD-L1 antibody, GPX4 antibody, xCT antibody, clusters of differentiation 31 (CD31) antibody, clusters of differentiation 44 (CD44) antibody, E-cadherin antibody, N-cadherin antibody, matrix metalloproteinases-9 (MMP-9) antibody, CX3CL1 antibody, colony-stimulating factor 1 receptor (CSF-1R) antibody, clusters of differentiation16/32 (CD16/32) antibody, clusters of differentiation 206 (CD206) antibody, ionized calcium binding adaptor molecule 1 (Iba-1) antibody were bought from Abcam (London, England). CSF-1 antibody and C-X3-C motif chemokine receptor 1 (CX3CR1) antibody were bought from Proteintech (Beijing, China). β-actin antibody were bought from Affinity (Colorado, USA). GM-CSF was bought from Peprotech (Suzhou, China). Anti-mouse clusters of differentiation 11 c-allophycocyanin (CD11c-APC), anti-mouse clusters of differentiation 80-fluorescein (CD80-FITC), anti-mouse clusters of differentiation86-phycoerythrin (CD86-PE), anti-mouse clusters of differentiation 3-allophycocyanin (CD3-APC), anti-mouse clusters of differentiation 8-fluorescein isothiocyanate (CD8-FITC), anti-mouse interferon-γ-phycoerythrin-sulfo-cyanine7 (IFN-γ-PE-Cy7), anti-mouse clusters of differentiation4-phycoerythrin-sulfo-cyanine7 (CD4-PE-Cy7), anti-mouse clusters of differentiation 25-fluorescein isothiocyanate (CD25-FITC), anti-mouse forkhead box p3-phycoerythrin (FoxP3-PE) were obtained from Biolegend (California, USA). Enzyme linked immunosorbent assay (ELISA) kits were obtained from Cloud-Clone corp (Wuhan, China). GSH detection kit and H_2_O_2_ detection kit were bought from Solarbio (Beijing, China). Reactive oxygen species (ROS) detection kit was bought from Bestbio (Shanghai, China). Boron difluoride pyrrole fluorescent dyes-C11 (BODIPY-C11) staining solution was bought from ThermoFisher (Massachusetts, USA).

GL261 cells, HT-22 cells, BV2 cells, RAW264.7 cells and bEnd3 cells were purchased from CytoBiotech (Xi’an, China). TMZ-resistant GL261 cells were induced in our lab. 6 weeks old C57 mice and 8 weeks old Sprague-Dawley (SD) male rats were provided by the Experimental Animal Center of Air Force Medical University (Xi’an, China).

### Extraction of BV2 cell membrane

BV2 cells in logarithmic growth phase were collected and mixed with 3 mL low-osmotic lysate and 30 μL protease inhibitor. Then, BV2 cells suspension was immerged into liquid nitrogen. After BV2 cells suspension was frozen and thawed for 3 times, the cell lysate was centrifuged for 10 min at 4 ℃ (14,000 × *g*). The supernatant was discarded, and 3 mL sterilized deionized water was added into precipitate. The mixture was performed ultrasound for 2 min, and supernatant was collected by centrifuging mixture for 20 min at 4 ℃ (14,000 × g). Then BV2 cell membrane (M_-BV2_) was obtained by lyophilizing the supernatant.

### Preparation of Fe_3_O_4_-siPD-L1@M_-BV2_

Thiolated siPD-L1 (150 μL, 0.264 mg/mL), H_2_O_2_ (30%, 55 μL) and thiolated Fe_3_O_4_ nanoparticles (120 μL, 5 mg/mL) were added into enzyme-free EP tube, and the mixture was stirred for 1 h at room temperature to connect siPD-L1 with Fe_3_O_4_ nanoparticles by disulfide bond. The reaction mixture was centrifuged for 10 min at 4 ℃ (9000 × *g*), and the supernatant was discarded. The precipitate was washed with DEPC water for 6 times to completely remove free siPD-L1. After that, precipitate was re-suspended into PBS buffer to get Fe_3_O_4_-siPD-L1 suspension. The mass ratio between thiolated Fe_3_O_4_ and thiolated siPD-L1 was optimized by agarose gel electrophoresis. Scramble siPD-L1 was used to prepare Fe_3_O_4_-siNC nanoparticle by using the same method in the preparation of Fe_3_O_4_-siPD-L1. Finally, 3 mL sterilized deionized water containing 10 mg M_-BV2_ was added into Fe_3_O_4_-siPD-L1 suspension. After being performed ultrasonic for 1 min, the mixture solution was incubated at 37 ℃ for 10 min. The mixture solution was extruded through 0.4 μm polycarbonate membrane for 21 times to obtain Fe_3_O_4_-siPD-L1@M_-BV2_. Fe_3_O_4_-FAM was prepared by reacting Fe_3_O_4_-siPD-L1 with maleimide modified carboxyfluorescein (FAM-MAL). To prepare Fe_3_O_4_-FAM@M_-BV2_, M_-BV2_ was coated on the surface of Fe_3_O_4_-FAM by using the same method in the preparation of Fe_3_O_4_-siPD-L1@M_-BV2_.

### Characterization of Fe_3_O_4_-siPD-L1@M_-BV2_

Firstly, the morphology and element composition of Fe_3_O_4_-siPD-L1@M_-BV2_ were investigated by field emission transmission electron microscopy (TEM, FEI Talos F200X, Super-X, ThermoFisher, USA). Particle size, zeta potential and the stability of Fe_3_O_4_ nanoparticles and Fe_3_O_4_-siPD-L1@M_-BV2_ in deionized water, phosphate buffer solution (PBS) and fetal bovine serum (FBS, 10%) were determined by dynamic laser particle size analyzer (Delsa Nano C, Beckman, USA). Fe_3_O_4_-siPD-L1@M_-BV2_ dispersion was placed at 37 ℃ in a constant temperature and humidity incubator. After Fe_3_O_4_-siPD-L1 was labeled by FAM and M_-BV2_ was labeled by DiI, the membrane structure of Fe_3_O_4_-siPD-L1@M_-BV2_ was observed by laser scanning confocal microscope (LSCM, Nikon, Japan). The proteins maintained in Fe_3_O_4_-siPD-L1@M_-BV2_ were observed by SDS-PAGE [42]. Besides, proteins such as CX3CR1, CSF-1R, CX3CL1 and CSF-1 in Fe_3_O_4_-siPD-L1@M_-BV2_ were detected by western blot [37]. The hemolysis of Fe_3_O_4_-siPD-L1@M_-BV2_ was investigated by using rat erythrocytes [43]. The release of siPD-L1 from Fe_3_O_4_-siPD-L1@M_-BV2_ in GSH-containing solution was observed by gel retardation assay [44]. The stability of Fe_3_O_4_-siPD-L1@M_-BV2_ in RNase A-containing buffer was observed by gel retardation assay.

### The selective uptake of Fe_3_O_4_-FAM@M_-BV2_ by GL261/TR cell, HT-22 cell, BV2 cell and RAW264.7 cell

GL261/TR cells, HT-22 cells, BV2 cells and RAW264.7 cells in logarithmic growth phase were separately inoculated into different 24-well plates containing cover glass at density of 2 × 10^5^ cells/mL and incubated at 37 ℃ for 24 h. Four cover glass inoculated with different cells were transferred into one well of 6-well plate, and 2 mL of fresh serum-free dulbecco's modified eagle medium (DMEM) containing Fe_3_O_4_-FAM@M_-BV2_ (the equivalent Fe_3_O_4_ concentration was 200 μg/mL) was added into each well. Fe_3_O_4_-FAM was used as control. The cells were cultured for 1, 2 and 4 h, respectively. (1) The cells were collected and re-suspend in PBS. The uptake of Fe_3_O_4_-FAM@M_-BV2_ by GL261/TR cell, HT-22 cell, BV2 cell and RAW264.7 cell was detected by flow cytometer (Beckman, A00-1–1102, USA). (2) The cell culture medium was discarded, and the cells were fixed with 4% paraformaldehyde for 10 min. The cells were washed with PBS for 3 times. Then cells were stained with 4’,6-diamidino-2-phenylindole (DAPI) solution (0.5 μg/mL) for 10 min. After the cells were washed with PBS for 3 times, the uptake of Fe_3_O_4_-FAM@M_-BV2_ by GL261/TR cell, HT-22 cell, BV2 cell and RAW264.7 cell was observed by LSCM.

### The uptake mechanism of Fe_3_O_4_-FAM@M_-BV2_ by GL261/TR cell

(1) Fresh serum-free DMEM containing Fe_3_O_4_-FAM@M_-BV2_ (the equivalent Fe_3_O_4_ concentration was 200 μg/mL) was co-incubated with CX3CR1 antibody (1 μg/mL) for 2 h at 37 ℃, and then the mixture was added into a 24-well plate pre-inoculated with GL261/TR cells on a cover glass. The cell was culture at 37 ℃ for 4 h. (2) Fresh serum-free DMEM containing CX3CL1 antibody (1 μg/mL) was added into a 24-well plate pre-inoculated with GL261/TR cells on a cover glass. After co-incubation at 37 ℃ for 2 h, fresh serum-free DMEM containing Fe_3_O_4_-FAM@M_-BV2_ (the equivalent Fe_3_O_4_ concentration was 200 μg/mL) was added into cell culture medium, and cell was cultured at 37 ℃ for 4 h. (3) Chlorpromazine solution (10 μg/mL), colchicine solution (800 μg/mL), methyl-β-cyclodextrin solution (5 μg/mL), and 2-deoxy-D-glucose (900 μg/mL) were added into 24-well plates pre-inoculated with GL261/TR cells on a cover glass. The cell was incubated at 37 ℃ for 2 h, and then fresh serum-free DMEM containing Fe_3_O_4_-FAM@M_-BV2_ (equivalent Fe_3_O_4_ concentration was 200 μg/mL) was added into cell culture medium. The cell was cultured at 37 ℃ for 4 h. (4) The GL261/TR cell on a cover glass was incubated at 4 ℃ for 1 h, and then fresh serum-free DMEM containing Fe_3_O_4_-FAM@M_-BV2_ (4 ℃, equivalent Fe_3_O_4_ concentration was 200 μg/mL) was added into cell culture medium. The cell was cultured at 4 ℃ for 4 h. After that, the cells were collected and re-suspend in PBS. The uptake of Fe_3_O_4_-FAM@M_-BV2_ by GL261/TR cell, HT-22 cell, BV2 cell and RAW264.7 cell was detected by flow cytometer. Besides, the cells were fixed with 4% paraformaldehyde for 10 min. The cells were washed with PBS for 3 times. Then cells were stained with DAPI solution (0.5 μg/mL) for 10 min. After the cells were washed with PBS for 3 times, the uptake of Fe_3_O_4_-FAM@M_-BV2_ by GL261/TR cells was observed by LSCM.

### The silencing effect of Fe_3_O_4_-siPD-L1@M_-BV2_ on PD-L1 protein in GL261 cells and GL261/TR cells

GL261 cells and GL261/TR cells in logarithmic growth phase were inoculated into 6-well plates at density of 5 × 10^5^ cells/mL per well and incubated at 37 ℃ for 24 h. The culture medium was replaced with fresh serum-free DMEM medium containing Fe_3_O_4_-siNC (150 nM scramble siPD-L1), siPD-L1@Lipo2000 (150 nM siPD-L1), Fe_3_O_4_-siPD-L1 (150 nM siPD-L1) and Fe_3_O_4_-siPD-L1@M_-BV2_ (150 nM siPD-L1) and cultured for 48 h. Then, cells were collected and proteins were extracted. Western blot was used to investigate the silencing effect of Fe_3_O_4_-siPD-L1@M_-BV2_ on PD-L1 protein in GL261 cells and GL261/TR cells.

### Effects of Fe_3_O_4_-siPD-L1@M_-BV2_ on the viability of GL261/TR cells

GL261/TR cells in logarithmic growth phase were inoculated into 96-well plates at density of 3 × 10^4^ cells/mL per well and incubated at 37 ℃ for 24 h. The culture medium was replaced with serum-free DMEM medium containing Fe_3_O_4_, Fe_3_O_4_-siPD-L1 and Fe_3_O_4_-siPD-L1@M_-BV2_ (equivalent Fe_3_O_4_ concentration was 10, 20, 50, 100, 200 μg/mL), and the cells were incubated at 37 ℃ for 48 h. 20 μL MTT solution (5 mg/mL) was added into each well and incubated at 37 ℃ for 4 h. The culture medium in well was discarded, and 150 μL DMSO was added into each well. The absorbance of each well was measured at 490 nm by enzyme linked immune-analyzer (Bio-Rad Laboratories, Inc. California, USA), and the cell survival rate was calculated.

MTT method was also used to illuminate whether Fe_3_O_4_-siPD-L1@M_-BV2_ could induce ferroptosis. Briefly, GL261/TR cells in logarithmic growth phase were inoculated into 96-well plates at density of 3 × 10^4^ cells/mL per well and incubated at 37 ℃ for 24 h. The culture medium was replaced with fresh serum-free DMEM medium containing Fe_3_O_4_-siPD-L1@M_-BV2_ (equivalent Fe_3_O_4_ concentration was 10, 20, 50, 100, 200 μg/mL) at the present of IFN- γ(10 ng/mL), ferrostatin1 (Fer-1, 10 μM) and deferoxamine (DFO, 100 μM). The cells were incubated at 37 ℃ for 48 h. 20 μL MTT solution (5 mg/mL) was added into each well, and cells were incubated at 37℃ for 4 h. The culture medium in well was discarded, and 150 μL DMSO was added into each well. The absorbance of each well was measured at 490 nm by enzyme linked immune-analyzer, and the cell survival rate was calculated.

### Staining of living and dead cells

GL261/TR cells in logarithmic growth phase were inoculated into 6-well plates at density of 5 × 10^5^ cells/mL per well and incubated at 37 ℃ for 24 h. The culture medium was replaced with fresh serum-free DMEM medium containing Fe_3_O_4_-siNC, Fe_3_O_4_, Fe_3_O_4_-siPD-L1, Fe_3_O_4_-siPD-L1@M_-BV2_, Fe_3_O_4_-siPD-L1@M_-BV2_ + Fer-1, Fe_3_O_4_-siPD-L1@M_-BV2_ + DFO and Fe_3_O_4_-siPD-L1@M_-BV2_ + IFN-γ. The equivalent Fe_3_O_4_ concentration was 200 μg/mL. The concentration of IFN-γ, Fer-1 and DFO was 10 ng/mL, 10 μM and 100 μM, respectively. Cells were incubated at 37 ℃ for 48 h. The cells were collected and washed twice with 1 × assay buffer. Then, 1 mL staining solution was used to re-suspended cells, and cell suspension was incubated at 37 ℃ for 20 min. After centrifugation (2000 × g, 5 min), the supernatant was discarded, and the cells were re-suspended in PBS. The living and dead cells was observed under a fluorescence microscope (Nikon, Japan).

### Effects of Fe_3_O_4_-siPD-L1@M_-BV2_ on expression of GPX4 and xCT in GL261/TR cells

GL261/TR cells in logarithmic growth phase were inoculated into 6-well plates at density of 5 × 10^5^ cells/mL per well and incubated at 37 ℃ for 24 h. The culture medium was replaced with fresh serum-free DMEM medium containing IFN-γ, Fe_3_O_4_, Fe_3_O_4_-siPD-L1, Fe_3_O_4_-siPD-L1@M_-BV2_, Fe_3_O_4_-siPD-L1@M_-BV2_ + Fer-1, Fe_3_O_4_-siPD-L1@M_-BV2_ + DFO and Fe_3_O_4_-siPD-L1@M_-BV2_ + IFN-γ. The equivalent Fe_3_O_4_ concentration was 200 μg/mL. The concentration of IFN-γ, Fer-1 and DFO was 10 ng/mL, 10 μM and 100 μM, respectively. The cells were incubated at 37 ℃ for 48 h. Then, cells were collected and proteins were extracted. Western blot was used to detect protein expression of GPX4 and xCT in GL261/TR cells.

### Effects of Fe_3_O_4_-siPD-L1@M_-BV2_ on GSH and H_2_O_2_ level in GL261/TR cells

GL261/TR cells in logarithmic growth phase were inoculated into cell culture bottle (25 cm^2^) at density of 5 × 10^5^ cells/mL and incubated at 37 ℃ for 24 h. The culture medium was replaced with fresh serum-free DMEM medium containing Fe_3_O_4_-siNC, Fe_3_O_4_, Fe_3_O_4_-siPD-L1, Fe_3_O_4_-siPD-L1@M_-BV2_, Fe_3_O_4_-siPD-L1@M_-BV2_ + Fer-1, Fe_3_O_4_-siPD-L1@M_-BV2_ + DFO and Fe_3_O_4_-siPD-L1@M_-BV2_ + IFN-γ. The equivalent Fe_3_O_4_ concentration was 200 μg/mL. The concentration of IFN-γ, Fer-1 and DFO was 10 ng/mL, 10 μM and 100 μM, respectively. After the cells were incubated at 37 ℃ for 48 h, the cells were collected. The cells were frozen and thawed for 3 times. The cell lysate was centrifuged for 10 min (8000 × *g*, 15 min), and the supernatant was collected. The concentration of GSH and H_2_O_2_ in the supernatant was respectively detected by using GSH and H_2_O_2_ detection kit.

### Effects of Fe_3_O_4_-siPD-L1@M_-BV2_ on ROS and lipid peroxidation level in GL261/TR cells

GL261/TR cells in logarithmic growth phase were inoculated into 6-well plates at density of 5 × 10^5^ cells/mL per well and incubated at 37 ℃ for 24 h. The culture medium was replaced with fresh serum-free DMEM medium containing Fe_3_O_4_-siNC, Fe_3_O_4_, Fe_3_O_4_-siPD-L1, Fe_3_O_4_-siPD-L1@M_-BV2_, Fe_3_O_4_-siPD-L1@M_-BV2_ + Fer-1, Fe_3_O_4_-siPD-L1@M_-BV2_ + DFO and Fe_3_O_4_-siPD-L1@M_-BV2_ + IFN-γ. The equivalent Fe_3_O_4_ concentration was 200 μg/mL. The concentration of IFN-γ, Fer-1 and DFO was 10 ng/mL, 10 μM and 100 μM, respectively. The cells were incubated at 37 ℃ for 24 h. The cells were washed with serum-free DMEM medium for 3 times. (1) For detection ROS level, 1 mL of DHE dye solution (diluted 1000 times with serum-free DMEM medium) was added into each well. The cells were incubated at 37 ℃ for 1 h. Then cells were fixed with 4% paraformaldehyde and stained with DAPI solution (0.5 μg/mL). After the cells were washed with PBS for 3 times, the ROS in GL261/TR cells was observed under fluorescence microscope. (2) For detection lipid peroxidation level, 1 mL of C11 BODIPY dye solution (diluted 1000 times with serum-free DMEM medium) was added into each well. The cells were incubated at 37 ℃ for 0.5 h. Then cells were fixed with 4% paraformaldehyde and stained with DAPI solution (0.5 μg/mL). After the cells were washed with PBS for 3 times, the lipid peroxidation (LPO) in GL261/TR cells was observed under fluorescence microscope.

### Effects of Fe_3_O_4_-siPD-L1@M_-BV2_ on DC cell maturation in vitro

Femur and tibia of C57 male mice were isolated and immersed in 75% ethanol for 5 min. Then, femur and tibia were immersed in serum-free roswell park memorial institute 1640 medium (RPMI1640). The ends of the femur and tibia were cut off with scissors, and the bone marrow cells were rinsed out from the femur and tibia by using serum-free RPMI1640 medium. The culture medium containing bone marrow cells was filtered (70 μm, BioFIL). The filtrate was centrifuged (1000×*g*, 3 min), and supernatant was discard. The cells were re-suspended with red blood cell lysate (R1010, Solarbio). After the cell suspension was placed at room temperature for 1.5 min, RPM1640 complete medium was added. The supernatant was discarded by centrifugation (1000×*g*, 3 min), and the cells were re-suspended by RPM1640 complete medium containing GM-CSF (20 ng/mL). The cells were inoculated into 6-well plates at a density of 3×10^6^ cells/mL in each well (1 mL). 3 days later, 1 mL of RPMI1640 complete medium containing GM-CSF (20 ng/mL) was added into each well and cultured for another 2 days.

2 mL of cell culture medium containing Fe_3_O_4_, Fe_3_O_4_-siNC, Fe_3_O_4_-siPD-L1, Fe_3_O_4_-siPD-L1@M_-BV2_, Fe_3_O_4_-siPD-L1@M_-BV2_+IFN-γ, Fe_3_O_4_-siPD-L1@M_-BV2_+DFO, and Fe_3_O_4_-siPD-L1@M_-BV2_+Fer-1 (the equivalent Fe_3_O_4_ concentration was 200 μg/mL, the concentration of IFN-γ, Fer-1 and DFO was 10 ng/mL, 10 μM and 100 μM, respectively) was added into transwell donor chamber planted GL261/TR cells, respectively. After incubation for 6 h, donor chamber was transferred to a 6-well plate inoculated with DC cells at the bottom and cultured for 24 h. DC cells were collected and re-suspended with PBS. APC-CD11c antibody (0.2 mg/mL), FITC-CD80 antibody (0.5 mg/mL) and PE-CD86 antibody (0.2 mg/mL) were added into cell culture medium, and cell was incubated at 4 ℃ for 30 min in dark room. The cells were collected and re-suspended with 200 μL PBS. The proportion of CD11c^+^, CD86^+^ and CD80^+^ DCs was detected by flow cytometer.

### Efficiency of Fe_3_O_4_-siPD-L1@M_-BV2_ transport across the in vitro BBB

bEnd3 cells in the logarithmic growth phase were inoculated into the transwell donor chamber at a density of 5 × 10^5^ cells/mL per well. Serum-free DMEM medium was added into the recipient chamber, and bEnd3 cells were cultured at 37 ℃. The complete medium was replaced every two days. The resistance between transwell donor chamber and recipient chamber was measured by using a resistance meter. When the resistance value exceeded 200 Ω/cm^2^, the in vitro BBB model was regarded to be successful established [45].

The transwell donor chamber was transferred into a 24-well plate inoculated with GL261/TR cells at the bottom. 400 μL of fresh serum-free DMEM medium containing Fe_3_O_4_-FAM, Fe_3_O_4_-FAM@M_-BV2_ were added into donor chamber (the equivalent Fe_3_O_4_ concentration was 200 μg/mL). 0.8 mL of DMEM medium was added into recipient chamber. At the same time, a group with a magnet outside of the 24-well plates (Fe_3_O_4_-FAM@M_-BV2_ + magnet) was designed. After cell was incubated for 1 h, 4 h, 8 h and 12 h, the resistance values between transwell donor chamber and recipient chamber was measured to evaluate the integrity of the in vitro BBB model. The fluorescence intensity of the medium in recipient chamber was determined by fluorescence spectrophotometer (HITACHI, F-2700, Japan), and the in vitro BBB transmission efficiency of Fe_3_O_4_-FAM@M_-BV2_ was calculated. Finally, GL261/TR cells at the bottom of recipient chamber were collected and re-suspend in PBS. The uptake of Fe_3_O_4_-FAM@M_-BV2_ by GL261/TR cell was detected by flow cytometer. Besides, GL261/TR cells at the bottom of recipient chamber were fixed with 4% paraformaldehyde and then were stained with DAPI solution (0.5 μg/mL). After the cells were washed with PBS for 3 times, the uptake of Fe_3_O_4_-FAM@M_-BV2_ by GL261/TR cells was observed by LSCM after it penetrated in vitro BBB.

### Effects of CX3CL1 and CSF-1 on the transport of Fe_3_O_4_-siPD-L1@M_-BV2_ across the in vitro BBB

(1) After the successful establishment of the in vitro BBB model, the transwell donor chamber was transferred into a 24-well plates inoculated with GL261/TR cells at the bottom, and 800 μL fresh serum-free DMEM was added into recipient chamber. (2) After the successful establishment of the in vitro BBB model, the transwell donor chamber was transferred into a 24-well plates without GL261/TR cells at the bottom, and 800 μL fresh serum-free DMEM containing CX3CL1 (200 ng/mL) or CSF-1 (100 ng/mL) was added into recipient chamber. After that, 400 μL of fresh serum-free DMEM containing Fe_3_O_4_-FAM@M_-BV2_ was added into transwell donor chamber (the equivalent Fe_3_O_4_ concentration was 200 μg/mL). The 24-well plates without GL261/TR cell and chemokine was used as the control. After incubation for 4 h, the fluorescence intensity of the culture medium in recipient chamber was measured by fluorescence spectrophotometer.

After the successful establishment of the in vitro BBB model, transwell donor chamber was transferred into a 24-well plate inoculated with GL261/TR cells. (1) Fe_3_O_4_-FAM@M_-BV2_ (the equivalent Fe_3_O_4_ concentration was 200 μg/mL) was incubated with fresh serum-free DMEM containing CX3CR1 or CSF-1R antibody for 2 h at 37 ℃, and then they were added into transwell donor chamber. 800 μL of serum-free DMEM was added into recipient chamber. (2) Fresh serum-free DMEM containing CX3CL1 or CSF-1 antibody was added into recipient chamber and incubated with GL261/TR cells at 37 ℃ for 2 h. After that, 400 μL of fresh serum-free DMEM containing Fe_3_O_4_-FAM@M_-BV2_ was added into donor chamber (the equivalent Fe_3_O_4_ concentration was 200 μg/mL). The concentration of antibody was 1 μg/mL. Fe_3_O_4_-FAM@M_-BV2_ without incubation with antibody was used as control. After incubation for 4 h, the fluorescence intensity of the culture medium in recipient chamber was measured by fluorescence spectrophotometer.

### Establishment of orthotopic drug-resistant GBM model in mice

Luciferase expressed GL261/TR cell (Luc-GL261/TR) in logarithmic growth phase were prepared as 2 × 10^7^/mL cell suspension. C57 mice were anesthetized and fixed on the operation table, and a micro-injector was inserted into the skull at the right front 2 mm of the intersection of sagittal suture and coronal suture. 5 μL of cell suspension was slowly injected into the brain with the brain stereotactic locator to establish orthotopic drug-resistant GBM model in vivo.

### Distribution and pharmacokinetics of Fe_3_O_4_-siPD-L1@M_-BV2_ in orthotopic drug-resistant GBM mice

On the 10th day after plantation of Luc-GL261/TR cell, luciferase substrate was intraperitoneally injected (150 mg/kg). 5 min post injection, the mice were anesthetized with isoflurane. The GBM growth was observed by bioluminescence imaging (IVIS Lumina II, Caliper, USA), and the mice that the volume of orthotopic drug-resistant GBM did not meet the requirements were excluded.

The GBM-bearing mice were randomly divided into 4 groups. siPD-L1, Fe_3_O_4_-siPD-L1, Fe_3_O_4_-siPD-L1@M_-BV2_ and Fe_3_O_4_-siPD-L1@M_-BV2_ + magnet (Additional file 1: Fig. S1) were injected into GBM-bearing mice via tail vein (siPD-L1 was labeled by Cy5, equivalent siPD-L1 dose was 0.3 mg/kg). 6 h and 12 h later, in vivo bioluminescence imaging was used to observe the fluorescence distribution in the whole body of orthotopic drug-resistant GBM mice. The integrated brain targeting efficiency was calculated with the following equation. The integrated brain targeting efficiency = (fluorescence intensity in brain organ/fluorescence intensity in whole body) × 100%. Blood, brain, heart, liver, spleen, lung and kidney tissues were collected at 0.08, 1, 3, 6, 12 and 24 h after drug administration. In vivo bioluminescence imaging was used to observe the distribution of Cy5 labeled Fe_3_O_4_-siPD-L1@M_-BV2_ in brain, heart, liver, spleen, lung and kidney. The brain tissue was immobilized in 4% paraformaldehyde for 24 h. After tissue was sectioned, DAPI was used to label the nuclei, CD31 antibody was used to label the tumor vessels, and the distribution of Fe_3_O_4_-siPD-L1@M_-BV2_ in drug-resistant glioma tissue was observed by LSCM. Fluorescence spectrophotometer was used to detect the Cy5 labeled Fe_3_O_4_-siPD-L1@M_-BV2_ concentration in plasma samples. The GBM tissue was isolation from brain tissue. The GBM tissue and normal brain tissue were respectively ground with 0.8 mL PBS buffer in an ice bath, and the tissue homogenate was centrifuged (9000 × g, 15 min, 4 ℃). The precipitation and supernatant were separated. (1) The concentration of Cy5 labeled Fe_3_O_4_-siPD-L1@M_-BV2_ in extracellular fluid of brain tissue was detected by fluorescence spectrophotometer. (2) The precipitation was ground with 0.6 mL RIPA cell lysate. The cell lysate was centrifuged (9000 × g, 20 min, 4 ℃). The intracellular Cy5 labeled Fe_3_O_4_-siPD-L1@M_-BV2_ concentration in GBM tissue was detected by fluorescence spectrophotometer. (3) The intracellular content of Fe^2+^ in GBM tissue was detected by using Perls stain kit. The precipitation was ground with 0.6 mL RIPA cell lysate. The cell lysate was centrifuged (9000 × g, 20 min, 4 ℃). 0.5 mL of supernatant was added into 0.5 mL NH_4_Fe(SO_4_)_2_ solution. The above mixture solution was shaken at room temperature for 30 min. Then, the absorbance value of mixture solution at 700 nm was detected by using UV spectrophotometer. After that, dilute nitric acid was added into mixture solution, and the absorbance value of mixture solution at 700 nm was detected again by using UV spectrophotometer. The content of Fe^2+^ can be calculated according to the change of absorbance value.

### The therapeutic effect of Fe_3_O_4_-siPD-L1@M_-BV2_ on orthotopic drug-resistant GBM in mice

On the 10th day after plantation of Luc-GL261/TR cell, luciferase substrate was intraperitoneally injected (150 mg/kg). 15 min later, the mice were anesthetized with isoflurane. The GBM growth was observed by bioluminescence imaging, and the mice that the volume of orthotopic drug-resistant GBM did not meet the requirement was excluded.

The GBM-bearing mice were randomly divided into 10 groups: normal saline group, TMZ group (44 mg/kg), Fe_3_O_4_ group, Fe_3_O_4_-siNC group (equivalent siNC dose was 0.3 mg/kg), Fe_3_O_4_-siPD-L1 group (equivalent siPD-L1 dose was 0.3 mg/kg), Fe_3_O_4_-siPD-L1@M_-BV2_ group (equivalent siPD-L1 dose was 0.3 mg/kg), Fe_3_O_4_-siPD-L1@M_-BV2_ group (equivalent siPD-L1 dose was 1 mg/kg), Fe_3_O_4_-siNC@M_-BV2_ (equivalent siNC dose was 1 mg/kg), Fe_3_O_4_-siNC@M_-BV2_ + magnet (equivalent siNC dose was 1 mg/kg) and Fe_3_O_4_-siPD-L1@M_-BV2_ + magnet group (equivalent siPD-L1 dose was 1 mg/kg). Different formulations were injected into GBM-bearing mice through tail vein once every three days, for a total of 4 times. (1) The body weight and the death of GBM-bearing mice were recorded. The survival curve was drawn, and the median survival time was calculated. (2) On 10, 17, 20 and 23 day after plantation of Luc-GL261/TR cell, the mice were intraperitoneally injected with luciferase substrate (150 mg/kg), the orthotopic GBM growth was observed by in vivo bioluminescence imaging. On the 24th day after plantation of Luc-GL261/TR cell, the GBM-bearing mice were sacrificed. Blood of GBM-bearing mice was collected and serum was separated. The contents of alanine aminotransferase (ALT), aspartate aminotransferase (AST), urea nitrogen (BUN) and creatinine (CREA) in serum were detected by automatic biochemical analyzer (Chemray 800, Shenzhen, China). The lymphocytes in orthotopic GBM tissue were isolated. The number of CD11c^+^CD86^+^CD80^+^ cells (mDCs), CD4^+^CD25^+^FoxP3^+^T cells (T_reg_ cell) and CD3^+^CD8^+^IFN-γ^+^T cells (T_eff_ cell) in orthotopic GBM tissue were detected by flow cytometer. The expressions of PD-L1, invasion-related proteins including E-cadherin, N-cadherin, CD44, matrix metalloproteinases-9 (MMP-9), tumor necrosis factor-α (TNF-α), ferroptosis-related proteins including GPX4 and xCT in orthotopic drug-resistant GBM tissue were detected by western blot. The TNF-α, IFN-γ, IL-6, IL-10 and IL-12 level in orthotopic drug-resistant GBM tissue was determined by ELISA kit. *H&E* staining, Ki67 staining and tdT-mediated dUTP nick-end labeling (TUNEL) staining was used to investigate the effects of Fe_3_O_4_-siPD-L1@M_-BV2_ on cell morphology, proliferation and apoptosis in orthotopic drug-resistant GBM tissue. The expression of CD16/32, CD206, Iba-1, GPX4 and PD-L1 in paraffin sections of orthotopic drug-resistant GBM tissue was observed by immunofluorescence staining method. Dihydroethidium (DHE) probe was used to stain ROS in frozen sections of drug-resistant GBM tissue. The content of GSH in orthotopic drug-resistant GBM tissue was determined by GSH detection kit. *H&E* staining was performed on heart, liver, spleen, lung and kidney tissues to investigate the in vivo toxicity of Fe_3_O_4_-siPD-L1@M_-BV2_ in GBM-bearing mice.

### Statistical analysis

All data are expressed as mean ± standard deviation. The statistics analysis of each group was performed by using one-way ANOVA with SPSS 26.0 statistical software. *p* < 0.05 was considered statistically significant.

## Results

### Characterization of Fe_3_O_4_-siPD-L1@M_-BV2_

**Fig. 1:**
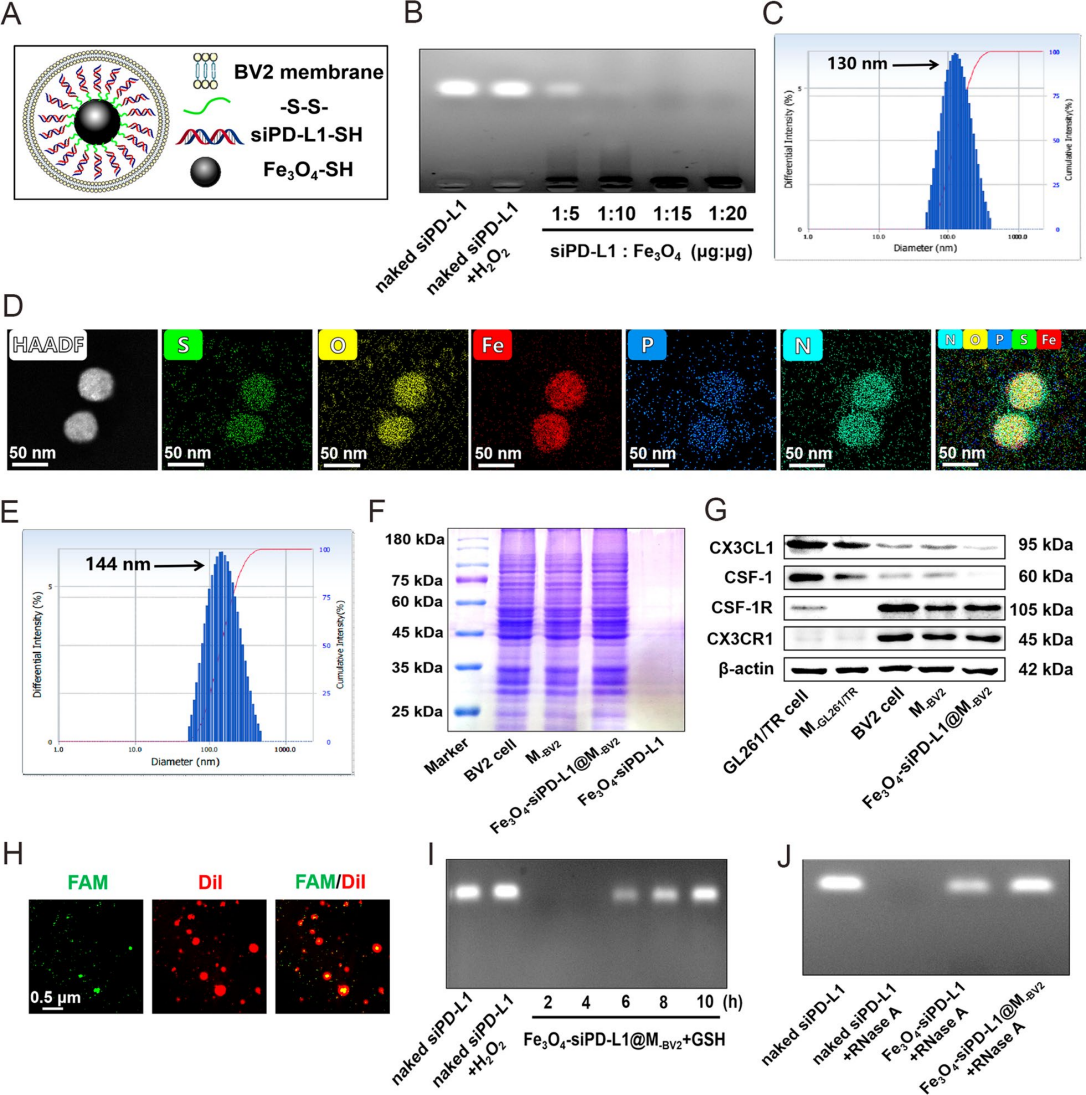
Characterization of Fe_3_O_4_-siPD-L1@M_-BV2_. **A** Diagram of the composition of Fe_3_O_4_-siPD-L1@M_-BV2_. **B** The optimized mass ratio between siPD-L1 and Fe_3_O_4_ screened by agarose gel electrophoresis. **C** Particle size distribution of Fe_3_O_4_. **D** Element mapping analysis diagram of Fe_3_O_4_-siPD-L1. **E** Particle size distribution of Fe_3_O_4_-siPD-L1@M_-BV2_. **F** SDS-PAGE protein analysis of Fe_3_O_4_-siPD-L1@M_-BV2_. **G** The protein expressions of CX3CL1, CSF-1, CX3CR1 and CSF-1R in GL261/TR cell, BV2 cell, GL261/TR cell membrane (M_-GL261/TR_), BV2 cell membrane (M_-BV2_) and Fe_3_O_4_-siPD-L1@M_-BV2_. **H** LSCM imaging of Fe_3_O_4_-siPD-L1@M_-BV2_. Green color (FAM) stands for Fe_3_O_4_-siPD-L1, and red color (DiI) stands for BV2 cell membrane. **I** GSH responsive of Fe_3_O_4_-siPD-L1@M_-BV2_ observed by agarose gel electrophoresis. **J** Stability of siPD-L1 in RNase A observed by agarose gel electrophoresis

The schematic diagram of Fe_3_O_4_-siPD-L1@M_-BV2_ is showing in Fig. 1A. The agarose gel electrophoresis experiment showed that siPD-L1 and Fe_3_O_4_ were completely connected when mass ratio between siPD-L1 and Fe_3_O_4_ was 1:15 (Fig. 1B). The average particle size of Fe_3_O_4_ was 130 nm (Fig. 1C). Mapping results showed that Fe_3_O_4_ contained O, Fe and S elements. Beside O, Fe and S, Fe_3_O_4_-siPD-L1 contained N and P elements, indicating that siPD-L1 was successfully connected with Fe_3_O_4_ (Fig. 1D; Additional file 1: Fig. S2A). The average particle size and zeta potential of Fe_3_O_4_-siPD-L1@M_-BV2_ was 144 nm and − 27 mV (Fig. 1E; Additional file 1: Fig. S2B). The protein bands of Fe_3_O_4_-siPD-L1@M_-BV2_ were consistent with that of BV2 cell membrane (M_-BV2_) (Fig. 1F), indicating that the proteins on BV2 cells membrane were well retained in Fe_3_O_4_-siPD-L1@M_-BV2_. Western blot results indicated that microglial chemokines CX3CL1 and CSF-1 were highly expressed in GL261/TR cells, and their receptors CX3CR1 and CSF-1R were retained in M_-BV2_ and Fe_3_O_4_-siPD-L1@M_-BV2_ (Fig. 1G). Moreover, LSCM experiment showed that red color of M_-BV2_ was completely merged with green color of Fe_3_O_4_ (Fig. 1H). The above results demonstrated that M_-BV2_ was successfully coated on the surface of Fe_3_O_4_-siPD-L1. Fe_3_O_4_-siPD-L1@M_-BV2_ remained stable within 7 days in PBS and water, and it was stable within 3 days in 10%FBS (Additional file 1: Fig. S2C). TEM results showed that the appearance of Fe_3_O_4_ and Fe_3_O_4_-siPD-L1@M_-BV2_ was spherical (Additional file 1: Fig. S2D, E). GSH promoted the release of siPD-L1 from Fe_3_O_4_-siPD-L1@M_-BV2_ in concentration-dependent manner (Fig. 1I). The naked siPD-L1 was rapidly degraded in RNase A solution, while Fe_3_O_4_-siPD-L1@M

_-BV2_ protected siPD-L1 from degradation by RNase A (Fig. 1J). Fe_3_O_4_-siPD-L1@M_-BV2_ did not cause hemolysis reaction (Additional file 1: Fig. S2F, G).

### Cellular uptake and gene silence efficiency of Fe_3_O_4_-FAM@M_-BV2_

**Fig. 2:**
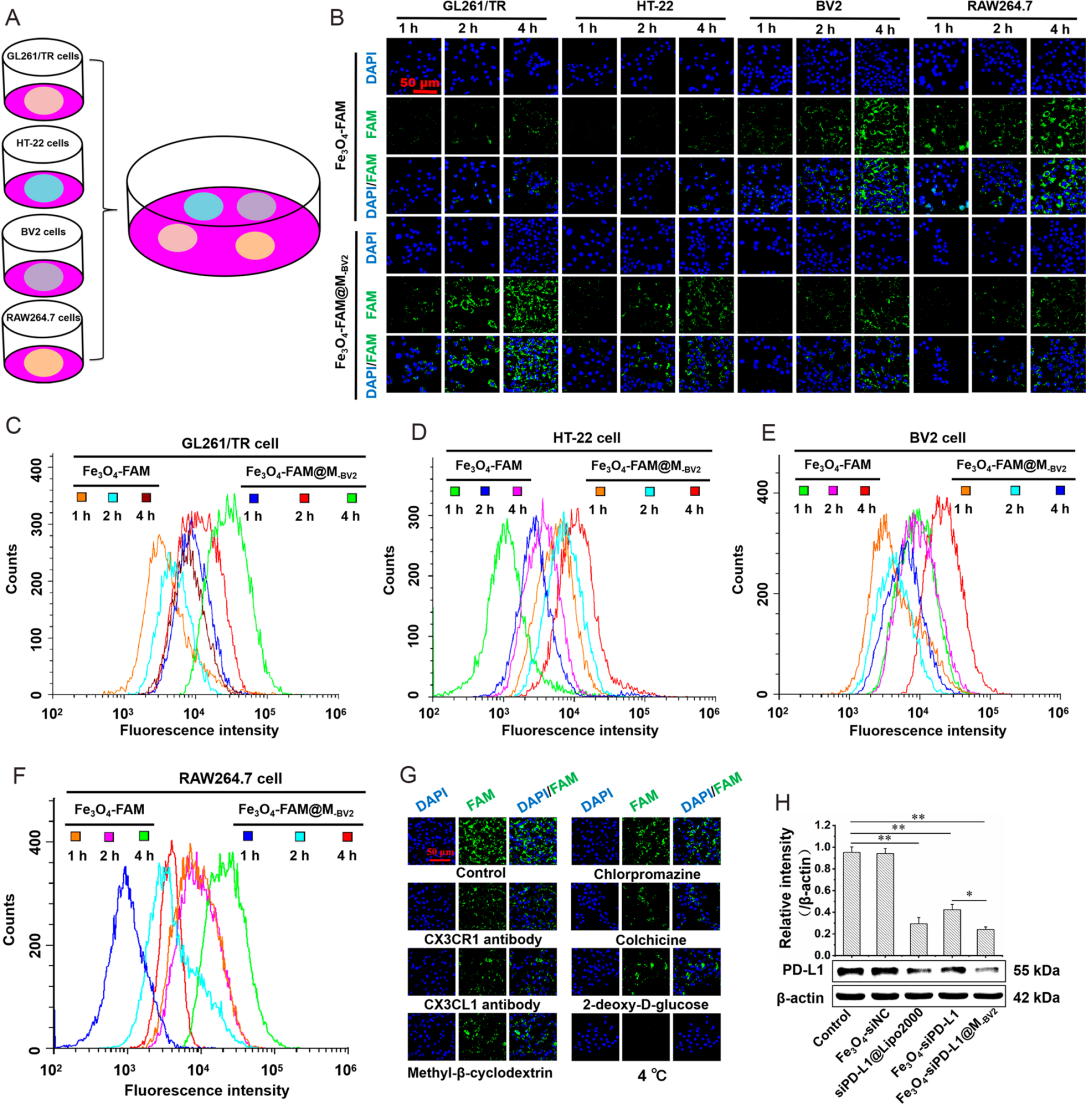
Uptake of Fe_3_O_4_-FAM@M_-BV2_ by GL261/TR cell. **A** Schematic diagram of co-culture of GL261/TR cells, HT-22 cells, BV2 cells and RAW264.7 cells in a petri dish. **B** The uptake of Fe_3_O_4_-FAM@M_-BV2_ by co-cultured GL261/TR cells, HT-22 cells, BV2 cells and RAW264.7 cells. **C**–**F** The typical flow cytometer diagrams of Fe_3_O_4_-FAM@M_-BV2_ uptake by co-cultured GL261/TR cells, HT-22 cells, BV2 cells and RAW264.7 cells. **G** Uptake of Fe_3_O_4_-FAM@M_-BV2_ by GL261/TR cells in the presence of different uptake inhibitors observed by LSCM. **H** The silence effect of Fe_3_O_4_-siNC, siPD-L1@Lipo2000, Fe_3_O_4_-siPD-L1 and Fe_3_O_4_-siPD-L1@M_-BV2_ on PD-L1 protein in GL261/TR cells (n = 3, mean ± SD, ^*^P < 0.05, ^**^P < 0.01)

GL261/TR cells, HT-22 cells, BV2 cells and RAW264.7 cells at logarithmic growth stage were respectively planted into 24-well plates containing cover glass at the same density. Cells were incubated at 37 ℃ for 24 h, the densities of the four cells were basically the same (Additional file 1: Fig. S3). When GL261/TR cells, HT-22 cells, BV2 cells and RAW264.7 cells were co-cultured in a petri dish (Fig. 2A), they took up Fe_3_O_4_-FAM@M_-BV2_ in a time-dependent manner. BV2 cells and RAW264.7 cells took up more amount of Fe_3_O_4_-FAM than Fe_3_O_4_-FAM@M_-BV2_. As compared with Fe_3_O_4_-FAM, more amount of Fe_3_O_4_-FAM@M_-BV2_ was taken up by GL261/TR cells. Moreover, GL261/TR cells took up much more amount of Fe_3_O_4_-FAM@M_-BV2_ than HT-22 cells did (Fig. 2B–F; Additional file 1: Fig. S4). This indicated that M_-BV2_ coating significantly reduced the uptake of Fe_3_O_4_-FAM@M_-BV2_ by BV2 cells and RAW264.7 cells. Fe_3_O_4_-FAM@M_-BV2_ was specifically taken up by GL261/TR cells. Furthermore, low temperature and 2-deoxy-D-glucose pretreatment markedly reduced the uptake of Fe_3_O_4_-FAM@M_-BV2_ by GL261/TR cells, indicating that the endocytosis of Fe_3_O_4_-FAM@M_-BV2_ by GL261/TR cells required energy supply. Colchicine significantly inhibited the uptake of Fe_3_O_4_-FAM@M_-BV2_ by GL261/TR cells, suggesting that Fe_3_O_4_-FAM@M_-BV2_ was taken by GL261/TR cells mainly through macropinocytosis. CX3CR1 antibody and CX3CL1 antibody also significantly reduced the uptake of Fe_3_O_4_-FAM@M_-BV2_ by GL261/TR cells, indicating that CX3CR1 and CX3CL1 were involved in the uptake of Fe_3_O_4_-FAM@M_-BV2_ by GL261/TR cells (Fig. 2G, Additional file 1: Fig. S5). After nanoparticle was taken up by GL261/TR cells, the silence efficiency of siPD-L1@Lipo2000, Fe_3_O_4_-siPD-L1 and Fe_3_O_4_-siPD-L1@M_-BV2_ on PD-L1 protein in GL261/TR cells were 69.18%, 58.74% and 74.81%, respectively (Fig. 2H). The similar results were observed in GL261 cells (Additional file 1: Fig. S6).

### The ferroptosis induced by Fe_3_O_4_-siPD-L1@M_-BV2_

**Fig. 3:**
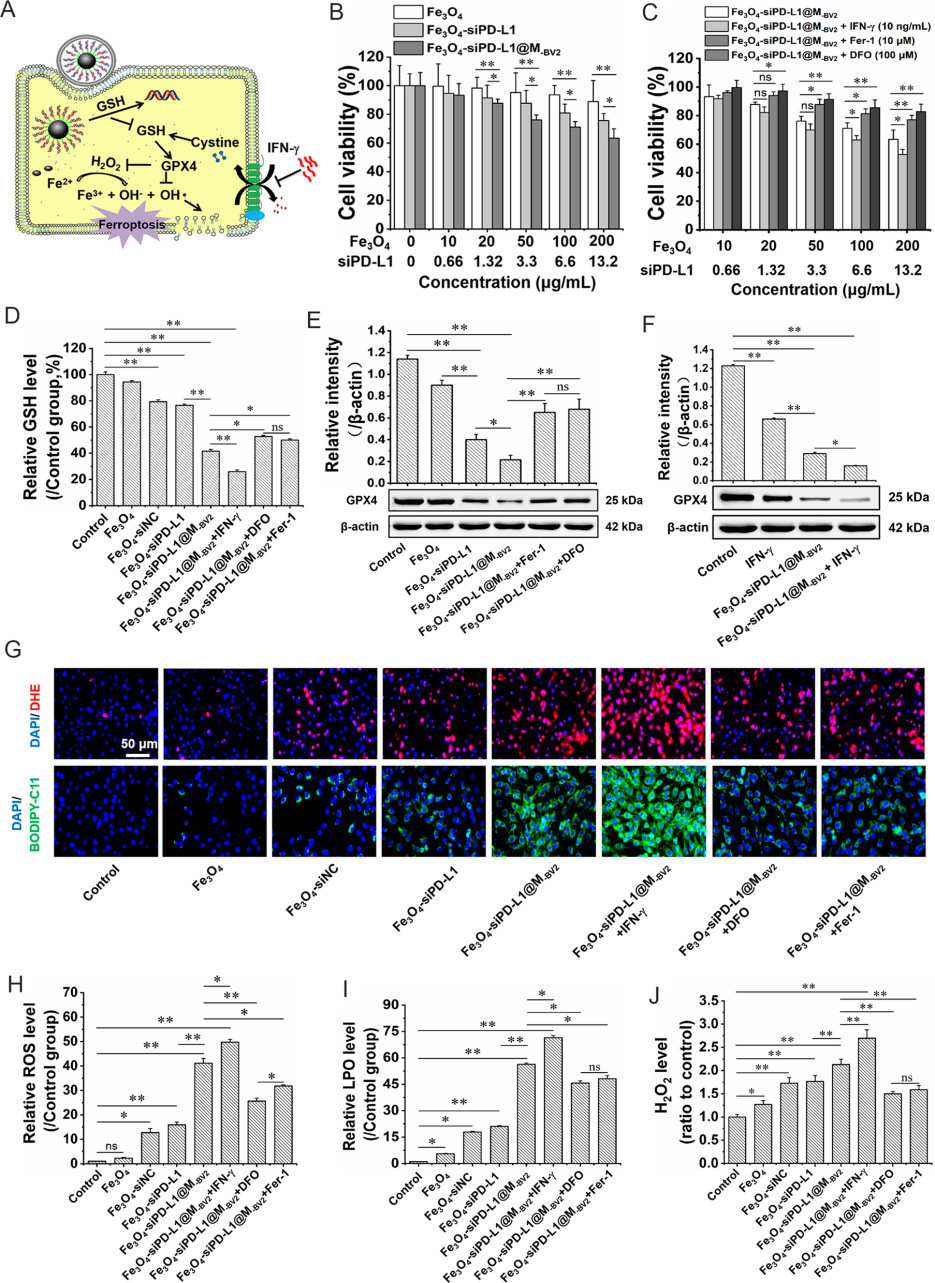
The ferroptosis induced by Fe_3_O_4_-siPD-L1@M_-BV2_ in GL261/TR cells. **A** Mechanism of ferroptosis. **B** Effect of Fe_3_O_4_-siPD-L1@M_-BV2_ on the viability of GL261/TR cells. **C** Effect of Fe_3_O_4_-siPD-L1@M_-BV2_ on the viability of GL261/TR cells in the presence of IFN-γ, Fer-1 and DFO. **D** Effect of Fe_3_O_4_-siPD-L1@M_-BV2_ on GSH level in GL261/TR cells. **E** Effect of Fe_3_O_4_-siPD-L1@M_-BV2_ on the protein expression of GPX4 in GL261/TR cells in the presence of Fer-1 and DFO. **F** Effect of Fe_3_O_4_-siPD-L1@M_-BV2_ on the protein expression of GPX4 in GL261/TR cells in the presence of IFN-γ. **G** Effect of Fe_3_O_4_-siPD-L1@M_-BV2_ on ROS (marked with DHE, red color) and LPO (marked with BODIPY-C11, green color) level in GL261/TR cells. **H** Statistic analysis of effect of Fe_3_O_4_-siPD-L1@M_-BV2_ on ROS level in GL261/TR cells. **I** Statistic analysis of effect of Fe_3_O_4_-siPD-L1@M_-BV2_ on LPO level in GL261/TR cells. **J** Effect of Fe_3_O_4_-siPD-L1@M_-BV2_ on H_2_O_2_ level in GL261/TR cells. (n = 3, mean ± SD, ^*^P < 0.05, ^**^P < 0.01; ns: no significant difference)

The essence of ferroptosis is Fenton reaction, which is triggered by Fe^2+^ and H_2_O_2_ (Fig. 3A). Firstly, MTT assay showed that Fe_3_O_4_-siPD-L1 and Fe_3_O_4_-siPD-L1@M_-BV2_ significantly reduced the activity of GL261/TR cells in a concentration-dependent manner. As compared with Fe_3_O_4_-siPD-L1, Fe_3_O_4_-siPD-L1@M_-BV2_ displayed stronger inhibitory effect on the activity of GL261/TR cells (Fig. 3B). In the presence of IFN-γ, the cytotoxicity of Fe_3_O_4_-siPD-L1@M_-BV2_ on GL261/TR cell was significantly enhanced. However, in the existence of ferroptosis inhibitor such as Fer-1 and DFO, the cytotoxicity of Fe_3_O_4_-siPD-L1@M_-BV2_ on GL261/TR cell was markedly attenuated (Fig. 3C). The same results were observed by living and dead cells staining experiment (Additional file 1: Fig. S7). The above results confirmed that Fe_3_O_4_-siPD-L1@M_-BV2_ induced ferroptosis.

Secondly, Fe_3_O_4_-siPD-L1@M_-BV2_ significantly reduced GSH level in GL261/TR cells. In the existence of IFN-γ, the GSH level in GL261/TR cells was further decreased by Fe_3_O_4_-siPD-L1@M_-BV2_. However, in the presence of Fer-1 and DFO, the GSH depletion induced by Fe_3_O_4_-siPD-L1@M_-BV2_ was weakened in GL261/TR cells (Fig. 3D).

Thirdly, Fe_3_O_4_ had no significant effect on GPX4 protein expression in GL261/TR cells. Fe_3_O_4_-siPD-L1 and Fe_3_O_4_-siPD-L1@M_-BV2_ significantly reduced the protein expression of GPX4 in GL261/TR cells (Fig. 3E). In the existence of Fer-1 and DFO, the inhibitory effect of Fe_3_O_4_-siPD-L1@M_-BV2_ on GPX4 protein expression in GL261/TR cells was weakened. IFN-γ inhibited GPX4 and x-CT protein expression in GL261/TR cells, and the inhibitory effect of Fe_3_O_4_-siPD-L1@M_-BV2_ on GPX4 protein expression was enhanced in the presence of IFN-γ (Fig. 3F; Additional file 1: Fig. S8).

Finally, Fe_3_O_4_-siNC, Fe_3_O_4_-siPD-L1 and Fe_3_O_4_-siPD-L1@M_-BV2_ significantly increased ROS, LPO and H_2_O_2_ level in GL261/TR cells. Moreover, in the presence of IFN-γ, ROS, LPO and H_2_O_2_ level in GL261/TR cells were further increased by Fe_3_O_4_-siPD-L1@M_-BV2_. However, in the presence of Fer-1 and DFO, the increase of ROS, LPO and H_2_O_2_ in GL261/TR cells induced by Fe_3_O_4_-siPD-L1@M_-BV2_ was attenuated (Fig. 3G–J).

### The maturation of DC cell induced by Fe_3_O_4_-siPD-L1@M_-BV2_ in vitro

**Fig. 4:**
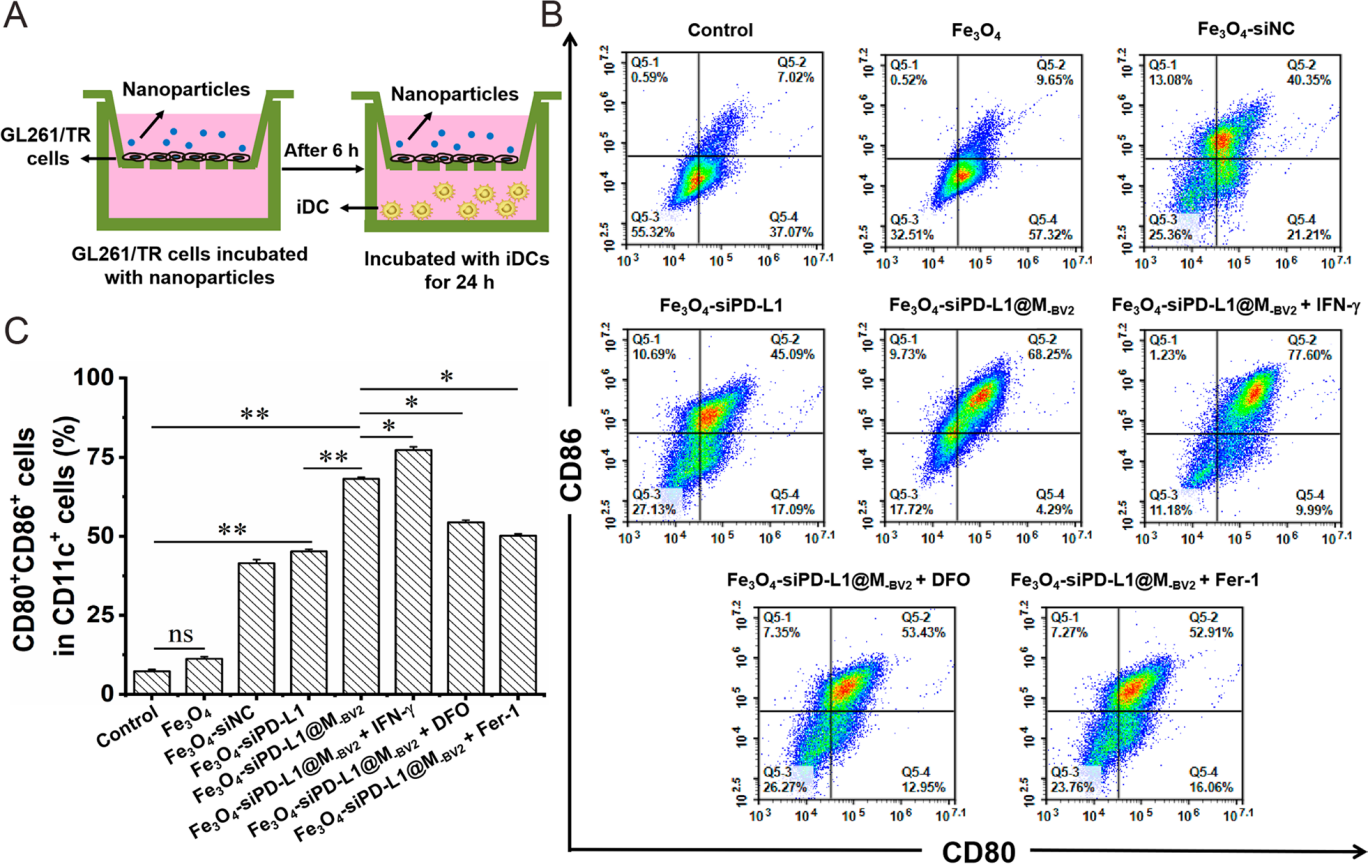
Effect of Fe_3_O_4_-siPD-L1@M_-BV2_ on the maturation of DC cell in vitro. **A** Schematic diagram of co-culture of GL261/TR cells and iDC cells. **B** The maturation of DC cells in vitro analyzed by flow cytometer. **C** Statistic analysis of the matured DC cell (n = 3, mean ± SD, ^*^P < 0.05, ^**^P < 0.01, ns: no significant difference)

Flow cytometer experiment showed that the proportion of CD11c^+^CD80^+^CD86^+^ cells in Fe_3_O_4_, Fe_3_O_4_-siPD-L1 and Fe_3_O_4_-siPD-L1@M_-BV2_ treatment groups was 9.67%, 45.26% and 68.2%, respectively, indicating that Fe_3_O_4_-siPD-L1 and Fe_3_O_4_-siPD-L1@M_-BV2_ significantly promoted the maturation of DC cells in vitro. As compared with Fe_3_O_4_-siPD-L1, Fe_3_O_4_-siPD-L1@M_-BV2_ facilitated the maturation of DC cells more strongly. In addition, the proportion of CD11c^+^CD80^+^CD86^+^ cells in Fe_3_O_4_-siPD-L1@M_-BV2_ + IFN-γ groups was increased to 77.31%, suggesting the effect of Fe_3_O_4_-siPD-L1@M_-BV2_ on inducing maturation of DC cell was further enhanced in the presence of IFN-γ. However, the proportion of CD11c^+^CD80^+^CD86^+^ cells in Fe_3_O_4_-siPD-L1@M_-BV2_ + DFO and Fe_3_O_4_-siPD-L1@M_-BV2_ + Fer-1 groups was 54.4% and 50.21%, respectively. This indicated that ferroptosis inhibitors significantly reduced the maturation of DC cell induced by Fe_3_O_4_-siPD-L1@M_-BV2_ (Fig. 4).

### Transport of Fe_3_O_4_-FAM@M_-BV2_ across in vitro BBB

**Fig. 5:**
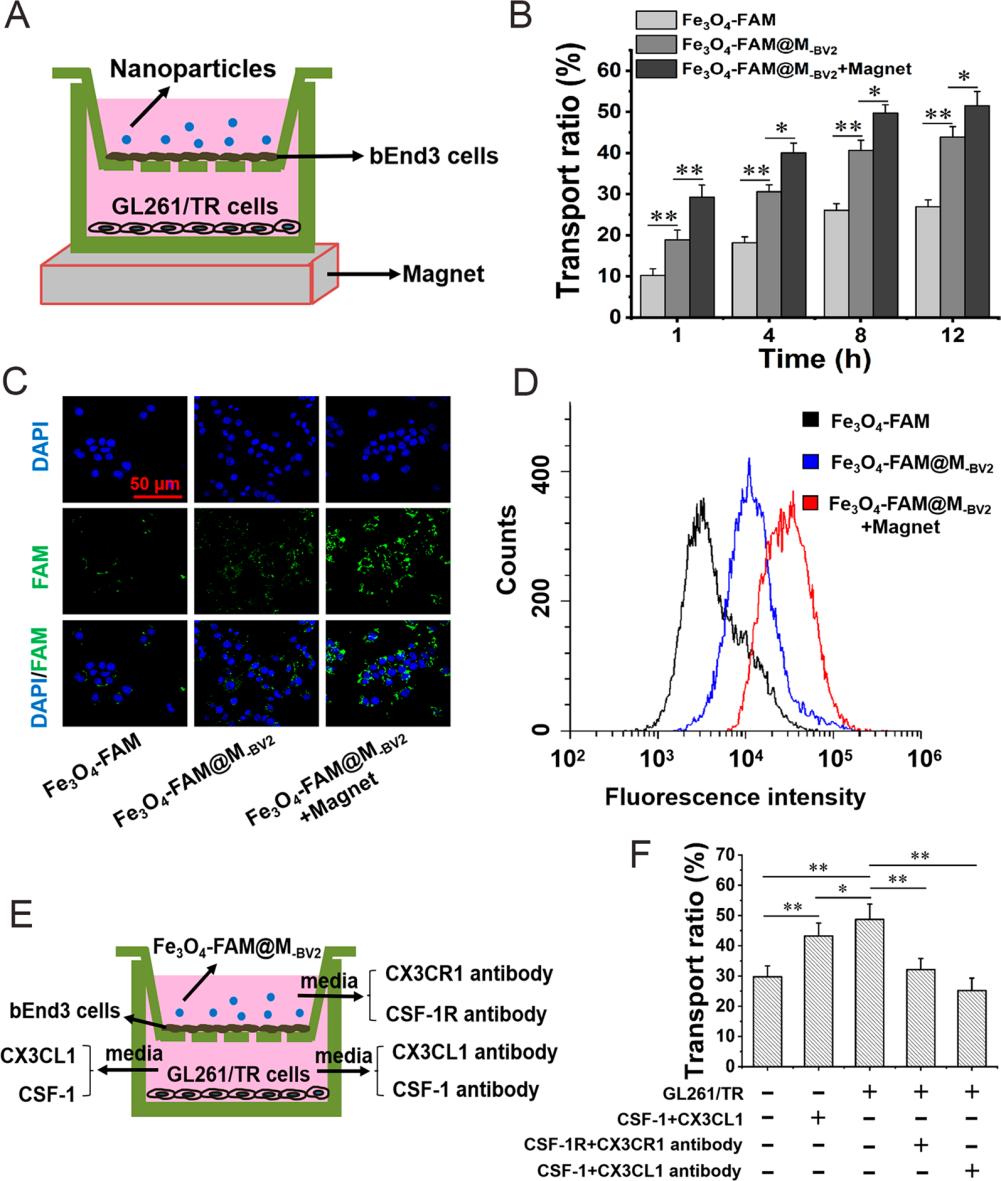
Transportation of Fe_3_O_4_-FAM@M_-BV2_ across in vitro BBB. **A** Schematic diagram of the in vitro BBB. **B** Transportation ratio of Fe_3_O_4_-FAM@M_-BV2_ across in vitro BBB. **C** The accumulation of Fe_3_O_4_-FAM@M_-BV2_ in GL261/TR cells after penetrating in vitro BBB detected by LCSM. **D** The typical flow cytometer diagrams of Fe_3_O_4_-FAM@M_-BV2_ accumulated in GL261/TR cells after Fe_3_O_4_-FAM@M_-BV2_ penetrated in vitro BBB. **E** Schematic diagram of mechanism study of Fe_3_O_4_-FAM@M_-BV2_ penetrating in vitro BBB. **F** The effect of CSF-1R, CX3CR1, CSF-1 and CX3CL1 on transportation ratio of Fe_3_O_4_-FAM@M_-BV2_ across the in vitro BBB. (n = 3, mean ± SD, ^*^P < 0.05, ^**^P < 0.01)

Within 12 h when drug is administered, there was no significant difference in resistance values between donor chamber and recipient chamber. It indicated that drug treatment did not damage the integrity of in vitro BBB model (Additional file 1: Fig. S9). As compared with Fe_3_O_4_-FAM, the transportation ratio of Fe_3_O_4_-FAM@M_-BV2_ across in vitro BBB was significantly higher, and it was further increased in the presence of external magnetic field (Fig. 5A, B). Compared with Fe_3_O_4_-FAM, the accumulation of Fe_3_O_4_-FAM@M_-BV2_ in GL261/TR cells was significantly increased after it penetrated in vitro BBB. In the presence of external magnetic field, the accumulation of Fe_3_O_4_-FAM@M_-BV2_ in GL261/TR cells was further increased after it penetrated in vitro BBB (Fig. 5C, D, Additional file 1: Fig. S10). Moreover, the transport ratio of Fe_3_O_4_-FAM@M_-BV2_ across in vitro BBB was significantly increased when GL261/TR cells were cultivated in recipient chamber in comparison with that without GL261/TR cells in recipient chamber. This suggested that GL261/TR cells facilitated the transport of Fe_3_O_4_-FAM@M_-BV2_ across in vitro BBB. When there were not GL261/TR cells in recipient chamber, the addition of CSF-1 and CX3CL1 in recipient chamber also significantly increased the transport ratio of Fe_3_O_4_-FAM@M_-BV2_ across in vitro BBB. After CSF-1 antibody and CX3CL1 antibody were added into recipient chamber, the transport ratio of Fe_3_O_4_-FAM@M_-BV2_ across in vitro BBB was significantly reduced. Moreover, when CSF-1R antibody and CX3CR1 antibody were added into donor chamber to block CSF-1R and CX3CR1 on the surface of Fe_3_O_4_-FAM@M_-BV2_, the transport ratio of Fe_3_O_4_-FAM@M_-BV2_ across in vitro BBB was also significantly decreased (Fig. 5E, F).

### Distribution and pharmacokinetics of Fe_3_O_4_-siPD-L1@M_-BV2_ in orthotopic drug-resistant GBM mice

**Fig. 6:**
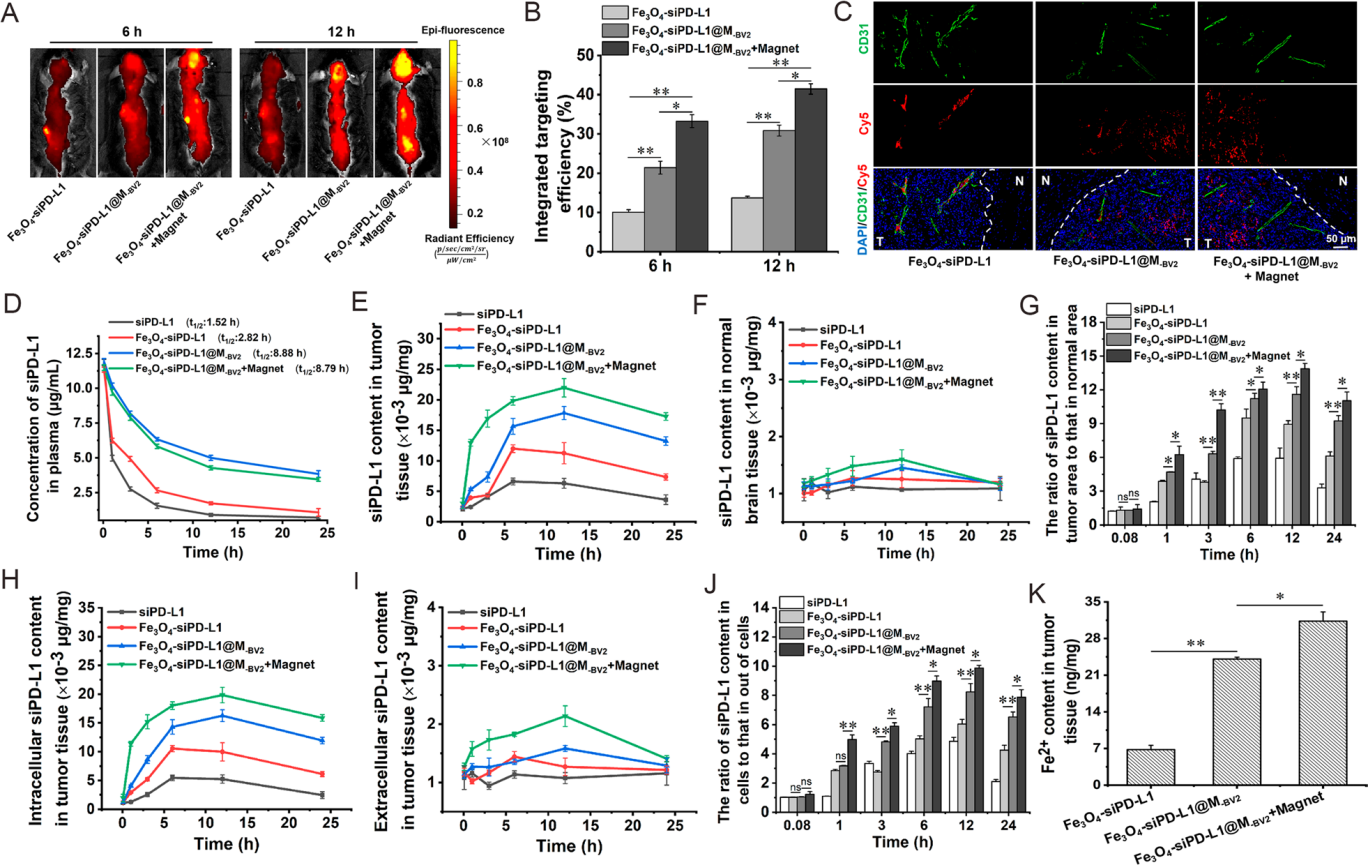
Distribution and pharmacokinetics of Fe_3_O_4_-siPD-L1@M_-BV2_ in orthotopic drug-resistant GBM mice. **A** The distribution of Fe_3_O_4_-siPD-L1@M_-BV2_ in orthotopic drug-resistant GBM mice observed by in vivo bioluminescence imaging. **B** The integrated brain targeting efficiency of Fe_3_O_4_-siPD-L1@M_-BV2_. **C** Distribution of Fe_3_O_4_-siPD-L1@M_-BV2_ in orthotopic drug-resistant GBM tissue observed by LSCM. CD31 staining: green color (stands for blood vessel). Cy5 staining: red color (stands for nanoparticle). T: tumor tissue; N: normal tissue. **D** Plasma siPD-L1 concentration–time curve in orthotopic drug-resistant GBM mice. **E** The content of siPD-L1 in orthotopic drug-resistant GBM tissue. **F** The content of siPD-L1 in normal brain tissue. **G** The siPD-L1 content ratio between in GBM tissue and in normal brain tissue. **H** The intracellular level of siPD-L1 in orthotopic drug-resistant GBM tissue. **I** The extracellular level of siPD-L1 in orthotopic drug-resistant GBM tissue. **J** The ratio of siPD-L1 content between in intracellular and in extracellular. **K** The intracellular Fe^2+^ level in orthotopic drug-resistant GBM tissue at 12 h after drug administration. (n = 3, mean ± SD, ^*^P < 0.05, ^**^P < 0.01, ns: no significant difference)

After intravenous administration of Fe_3_O_4_-siPD-L1@M_-BV2_, the distribution of Fe_3_O_4_-siPD-L1@M_-BV2_ in orthotopic drug-resistant GBM mice was observed by in vivo bioluminescence imaging. The results showed that as compared with Fe_3_O_4_-siPD-L1 group, fluorescence intensity in mice brain in Fe_3_O_4_-siPD-L1@M_-BV2_ group and Fe_3_O_4_-siPD-L1@M_-BV2_ + magnet group was significantly increased. Fe_3_O_4_-siPD-L1@M_-BV2_ + magnet group showed the strongest fluorescence intensity in mice brain (Fig. 6A; Additional file 1: Fig. S11). The integrated brain targeting efficiencies of Fe_3_O_4_-siPD-L1, Fe_3_O_4_-siPD-L1@M_-BV2_ and Fe_3_O_4_-siPD-L1@M_-BV2_ + magnet group at 12 h were 13.69%, 30.85% and 41.7%, respectively (Fig. 6B). Results of LSCM showed that a large amount of Fe_3_O_4_-siPD-L1@M_-BV2_ distributed in orthotopic drug-resistant GBM tissue, and little amount of Fe_3_O_4_-siPD-L1@M_-BV2_ distributed in the normal brain tissue. Moreover, Fe_3_O_4_-siPD-L1 mainly distributed in the blood vessels, while Fe_3_O_4_-siPD-L1@M_-BV2_ mainly distributed out of blood vessels in orthotopic drug-resistant GBM tissue (Fig. 6C), suggesting that Fe_3_O_4_-siPD-L1@M_-BV2_ penetrated deep region in GBM tissue.

Pharmacokinetic experiment showed that the half-life of siPD-L1 in plasma in naked siPD-L1, Fe_3_O_4_-siPD-L1, Fe_3_O_4_-siPD-L1@M_-BV2_ and Fe_3_O_4_-siPD-L1@M_-BV2_ + magnet groups was 1.52 h, 2.82 h, 8.88 h and 8.79 h, respectively (Fig. 6D). After administration of Fe_3_O_4_-siPD-L1@M_-BV2_ and Fe_3_O_4_-siPD-L1@M_-BV2_ + magnet, the content of siPD-L1 in orthotopic drug-resistant GBM tissue increased successively in comparison with naked siPD-L1 and Fe_3_O_4_-siPD-L1. Moreover, the content of siPD-L1 in orthotopic drug-resistant GBM tissue was significantly higher than that in normal brain tissue (Fig. 6E–G). As compared with the Fe_3_O_4_-siPD-L1 group, the intracellular level of siPD-L1 in drug-resistant GBM tissue was significantly increased in Fe_3_O_4_-siPD-L1@M_-BV2_ group and Fe_3_O_4_-siPD-L1@M_-BV2_ + magnet group (Fig. 6H–J). The intracellular level of Fe^2+^ in orthotopic drug-resistant GBM tissue was significantly increased in Fe_3_O_4_-siPD-L1@M_-BV2_ group and Fe_3_O_4_-siPD-L1@M_-BV2_ + magnet group as compared with that in Fe_3_O_4_-siPD-L1 group (Fig. 6K).

### The inhibitory effect of Fe_3_O_4_-siPD-L1@M_-BV2_ on the growth of orthotopic drug-resistant GBM in mice

**Fig. 7:**
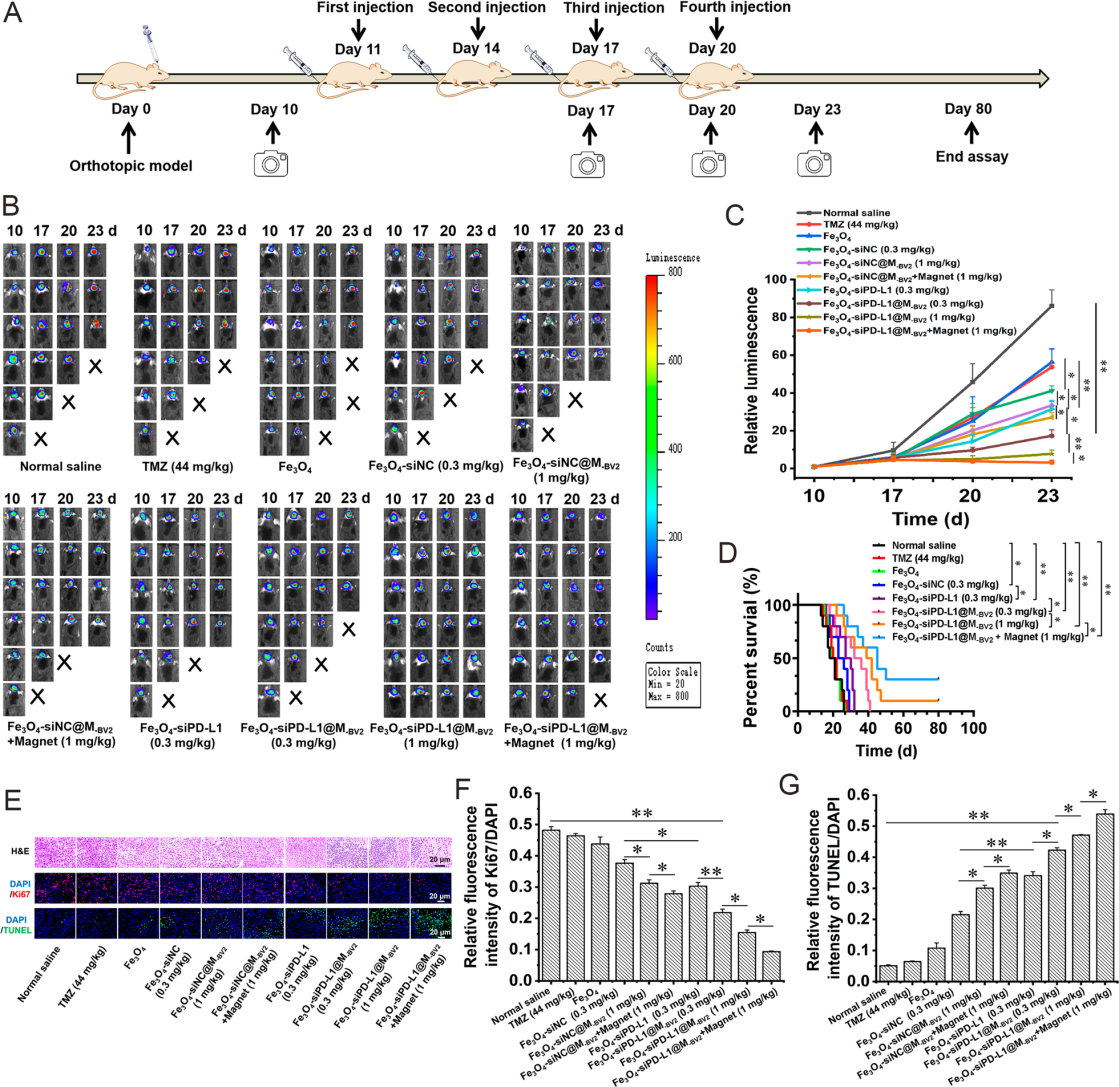
The therapeutic effect of Fe_3_O_4_-siPD-L1@M_-BV2_ on orthotopic drug-resistant GBM in mice. **A** Schematic diagram of drug administration and observation of therapeutic effect. **B** The inhibitory effect of Fe_3_O_4_-siPD-L1@M_-BV2_ on the growth of orthotopic drug-resistant GBM observed by in vivo bioluminescence imaging. **C** Statistical analysis of orthotopic drug-resistant GBM growth. (n = 6, mean ± SD, ^*^P < 0.05, ^**^P < 0.01). **D** Effects of Fe_3_O_4_-siPD-L1@M_-BV2_ on the survival time of orthotopic drug-resistant GBM mice. (n = 10, mean ± SD, ^*^P < 0.05, ^**^P < 0.01). **E** *H&E*, Ki67 and TUNEL staining of orthotopic drug-resistant GBM tissue. **F** Semi-quantitative analysis of Ki67 expressed in orthotopic drug-resistant GBM tissue. (n = 3, mean ± SD, ^*^P < 0.05, ^**^P < 0.01). **G** Semi-quantitative analysis of TUNEL positive cells in orthotopic drug-resistant GBM tissue. (n = 3, mean ± SD, ^*^P < 0.05, ^**^P < 0.01)

In vivo bioluminescence imaging was used to dynamic observe the growth of orthotopic drug-resistant GBM (Fig. 7A). As compared with normal saline, Fe_3_O_4_-siNC and Fe_3_O_4_-siNC@M_-BV2_ significantly inhibited the growth of orthotopic drug-resistant GBM. As compared with Fe_3_O_4_-siNC and Fe_3_O_4_-siNC@M_-BV2_, Fe_3_O_4_**-**siPD-L1 and Fe_3_O_4_-siPD-L1@M_-BV2_ significantly inhibited the growth of orthotopic drug-resistant GBM. Fe_3_O_4_-siPD-L1@M_-BV2_ displayed much stronger inhibitory effect on the growth of orthotopic drug-resistant GBM in comparison with Fe_3_O_4_-siPD-L1, suggesting that ferroptosis combined with immunotherapy further inhibited the growth of orthotopic drug-resistant glioma. In addition, in the presence of external magnetic field, the inhibitory effect of Fe_3_O_4_-siPD-L1@M_-BV2_ on orthotopic drug-resistant GBM was further enhanced (Fig. 7B, C).

On 26th, 28th, 27th, 29th, 32th and 41th day after the plantation of Luc-GL261/TR cell, all normal saline, TMZ, Fe_3_O_4_, Fe_3_O_4_-siNC, Fe_3_O_4_-siPD-L1 and low-dose Fe_3_O_4_-siPD-L1@M_-BV2_ treated orthotopic drug-resistant GBM mice died in succession. However, on 60 days after the plantation of Luc-GL261/TR cell, the survival rate was 10% and 30% in high-dose Fe_3_O_4_-siPD-L1@M_-BV2_ group and high-dose Fe_3_O_4_-siPD-L1@M_-BV2_ + magnet group, respectively (Fig. 7D). The median survival time of normal saline, TMZ, Fe_3_O_4_, Fe_3_O_4_-siNC, Fe_3_O_4_-siPD-L1, low-dose Fe_3_O_4_-siPD-L1@M_-BV2_, high-dose Fe_3_O_4_-siPD-L1@M_-BV2_ and high-dose Fe_3_O_4_-siPD-L1@M_-BV2_ + magnet treated GBM mice was 17.68, 21.39, 18.61, 22.28, 26.94, 31.56, 38.72 and 45.00 days, respectively.

*H&E* staining of orthotopic drug-resistant GBM tissue presented a long oval shape and a large number of pathological nuclear mitosis in normal saline, TMZ and Fe_3_O_4_ treated group. Fe_3_O_4_-siPD-L1@M_-BV2_ decreased the number of heteromorphic nuclear cells and nuclear division in orthotopic drug-resistant GBM tissue. The nucleus was regular spherical and the intercellular space became blurred or even disappeared in Fe_3_O_4_-siPD-L1@M_-BV2_ + magnet treated group (Fig. 7E). Moreover, Fe_3_O_4_-siNC@M_-BV2_, Fe_3_O_4_-siNC@M_-BV2_ + magnet, Fe_3_O_4_-siPD-L1@M_-BV2_ and Fe_3_O_4_-siPD-L1@M_-BV2_ + magnet obviously inhibited Ki67 expression and increased the number of TUNEL positive cells in orthotopic drug-resistant GBM tissue as compared with normal saline, free TMZ and Fe_3_O_4_ (Fig. 7E–G). These results indicated that Fe_3_O_4_-siPD-L1@M_-BV2_ markedly inhibited proliferation of orthotopic drug-resistant GBM cells and promoted its apoptosis.

The body weight of orthotopic drug-resistant GBM mice in normal saline, TMZ, Fe_3_O_4_, Fe_3_O_4_-siNC, Fe_3_O_4_-siPD-L1 and low-dose Fe_3_O_4_-siPD-L1@M_-BV2_ group decreased gradually. The body weight of orthotopic drug-resistant GBM mice in high-dose Fe_3_O_4_-siPD-L1@M_-BV2_ group and Fe_3_O_4_-siPD-L1@M_-BV2_ + magnet group showed a trend of increasing (Additional file 1: Fig. S12), indicating the growth of orthotopic drug-resistant GBM was markedly blocked by Fe_3_O_4_-siPD-L1@M_-BV2_ and Fe_3_O_4_-siPD-L1@M_-BV2_ + magnet.

### Mechanisms of Fe_3_O_4_-siPD-L1@M_-BV2_ on inhibition the growth of orthotopic drug-resistant GBM

Firstly, Fe_3_O_4_-siPD-L1, Fe_3_O_4_-siPD-L1@M_-BV2_ and Fe_3_O_4_-siPD-L1@M_-BV2_ + magnet significantly increased the protein expression of E-cadherin and decreased the protein expression of TGF-β, MMP-9, CD44 and N-cadherin in orthotopic drug-resistant GBM tissue. (Additional file 1: Fig. S13), indicating Fe_3_O_4_-siPD-L1@M_-BV2_ inhibited the invasion and migration of orthotopic drug-resistant GBM cells through regulating invasion-related protein expression.

**Fig. 8:**
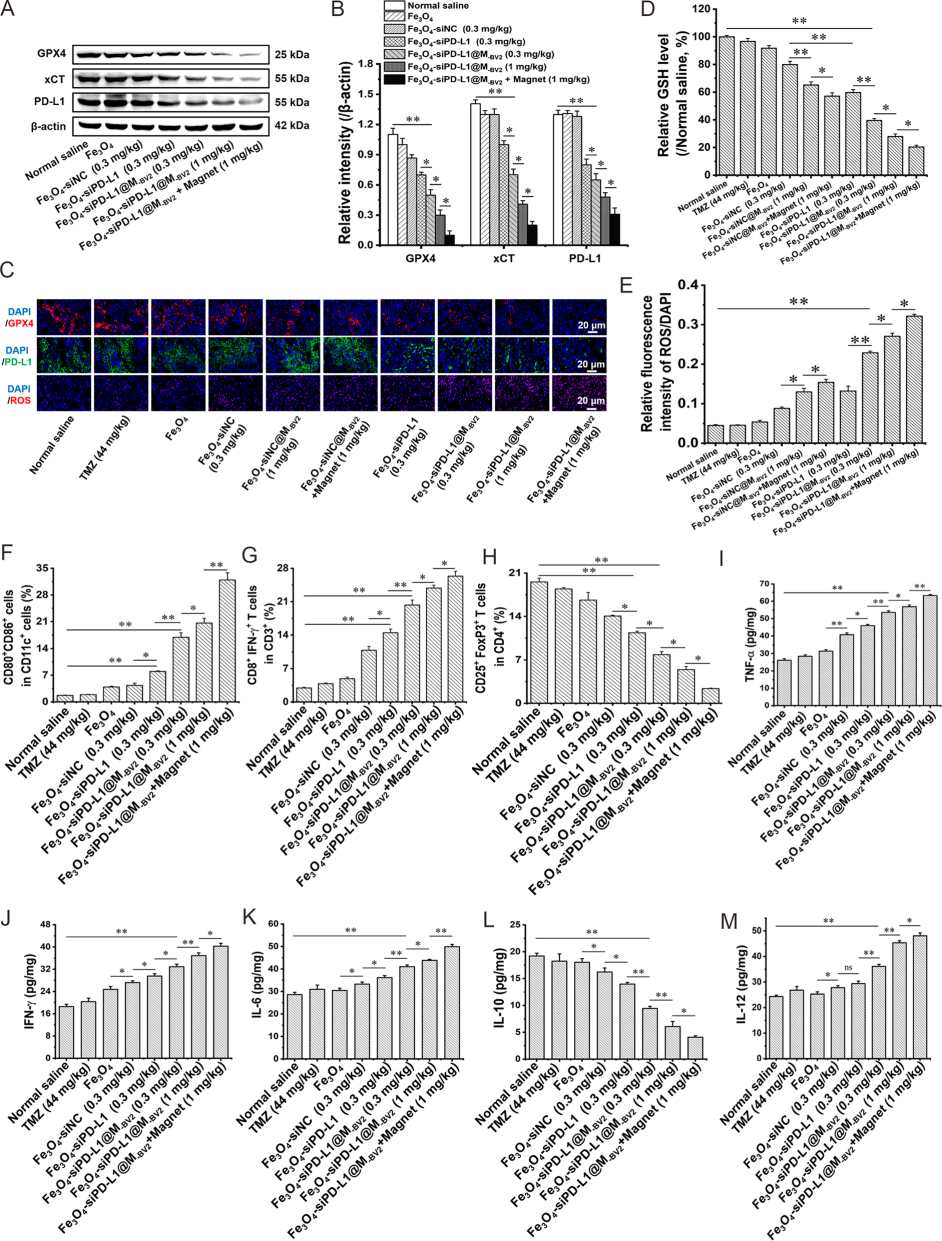
Effect of Fe_3_O_4_-siPD-L1@M_-BV2_ on immune microenvironment and ferroptosis in orthotopic drug-resistant GBM tissue. **A** The protein expression of PD-L1, xCT and GPX4 in orthotopic drug-resistant GBM tissue detected by western blot. **B** Statistic analysis of PD-L1, xCT and GPX4 protein expression. **C** The protein expression of PD-L1, GPX4 and ROS level in orthotopic drug-resistant GBM tissue observed by immunofluorescence staining. **D** The content of GSH in orthotopic drug-resistant GBM tissue. **E** Statistic analysis of ROS level in orthotopic drug-resistant GBM tissue. **F** The ratio of matured DC cell in orthotopic drug-resistant GBM tissue. **G**, **H** The proportion of T_eff_ cell and T_reg_ cell in orthotopic drug-resistant GBM tissue. **I**–**M** The content of TNF-α, IFN-γ, IL-6, IL-10 and IL-12 in orthotopic drug-resistant GBM tissue. (n = 3, mean ± SD, ^*^P < 0.05, ^**^P < 0.01)

Secondly, Fe_3_O_4_-siPD-L1@M_-BV2_ decreased the protein expression of PD-L1, x-CT and GPX4 in orthotopic drug-resistant GBM tissue in dose-dependent manner. As compared with Fe_3_O_4_-siPD-L1, Fe_3_O_4_-siPD-L1@M_-BV2_ reduced the protein expression of PD-L1, xCT and GPX4 more strongly. The protein expressions of PD-L1, xCT and GPX4 in orthotopic drug-resistant GBM tissue were less in Fe_3_O_4_-siPD-L1@M_-BV2_ + magnet group than that in Fe_3_O_4_-siPD-L1@M_-BV2_ group (Fig. 8A, B). At the same time, immunofluorescence staining was also used to investigate the effects of Fe_3_O_4_-siPD-L1@M_-BV2_ on the protein expression of GPX4 and PD-L1 in orthotopic drug-resistant GBM tissue, and the results were consistent with western blot experiment (Fig. 8C, Additional file 1: Fig. S14). In addition, Fe_3_O_4_-siPD-L1@M_-BV2_ dose-dependently reduced the content of GSH in orthotopic drug-resistant GBM tissue. The GSH content in orthotopic drug-resistant GBM tissue in Fe_3_O_4_-siPD-L1@M_-BV2_ + magnet group was lower in comparison with that in Fe_3_O_4_-siPD-L1@M_-BV2_ group (Fig. 8D). Meanwhile, Fe_3_O_4_-siPD-L1@M_-BV2_ dose-dependently increased the ROS level in orthotopic drug-resistant GBM tissue (Fig. 8C, E). Moreover, as compared with normal saline, Fe_3_O_4_-siNC and Fe_3_O_4_-siNC@M_-BV2_ also significantly inhibited the GPX4 expression, GSH and ROS level in orthotopic drug-resistant GBM tissue, indicating that Fe_3_O_4_-siNC and Fe_3_O_4_-siNC@M_-BV2_ induced ferroptosis and subsequently inhibited the growth of orthotopic drug-resistant glioma.

Thirdly, flow cytometer was used to investigate the effect of Fe_3_O_4_-siPD-L1@M_-BV2_ on the maturation of DC cell in orthotopic drug-resistant GBM tissue. The proportion of CD11c^+^CD80^+^CD86^+^ in normal saline, Fe_3_O_4_-siPD-L1, Fe_3_O_4_-siPD-L1@M_-BV2_ (0.3 mg/kg) and Fe_3_O_4_-siPD-L1@M_-BV2_ (1 mg/kg) treatment groups was 1.57%, 7.8%, 17.84%, 19.26%, respectively. As compared with Fe_3_O_4_-siPD-L1@M_-BV2_, the proportion of matured DC cells in Fe_3_O_4_-siPD-L1@M_-BV2_ + magnet group was significantly increased in orthotopic drug-resistant GBM tissue (Fig. 8F, Additional file 1: Fig. S15). Further study indicated that the proportion of CD3^+^CD8^+^IFN-γ^+^ T cells (T_eff_ cell) in orthotopic drug-resistant GBM tissue in normal saline, Fe_3_O_4_-siPD-L1, Fe_3_O_4_-siPD-L1@M_-BV2_ (0.3 mg/kg) and Fe_3_O_4_-siPD-L1@M_-BV2_ (1 mg/kg) treatment group was 3.10%, 13.43%, 19.25% and 23.1%, respectively. Fe_3_O_4_-siPD-L1@M_-BV2_ + magnet obviously increased the number of T_eff_ cell in orthotopic drug-resistant GBM tissue as compared with Fe_3_O_4_-siPD-L1@M_-BV2_ (Fig. 8G, Additional file 1: Fig. S16). The proportion of CD4^+^CD25^+^FoxP3^+^ T cells (T_reg_ cell) in orthotopic drug-resistant GBM tissue in normal saline, Fe_3_O_4_-siPD-L1, Fe_3_O_4_-siPD-L1@M_-BV2_ (0.3 mg/kg) and Fe_3_O_4_-siPD-L1@M_-BV2_ (1 mg/kg) treatment group was 18.76%, 11.05%, 7.24% and 5.29%, respectively. As compared with Fe_3_O_4_-siPD-L1@M_-BV2_, Fe_3_O_4_-siPD-L1@M_-BV2_ + magnet markedly reduced the number of T_reg_ cell in orthotopic drug-resistant GBM tissue (Fig. 8H, Additional file 1: Fig. S17).

**Fig. 9:**
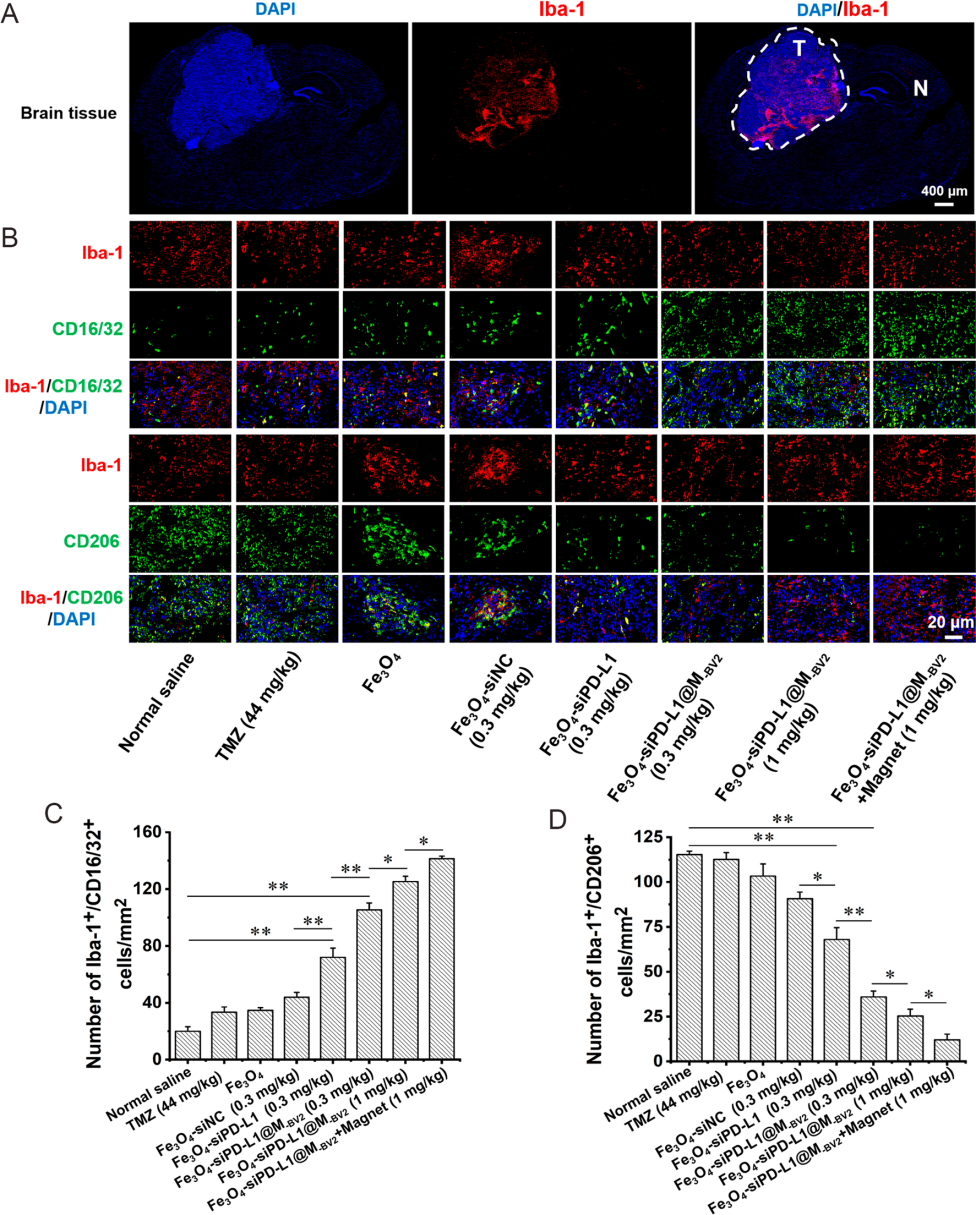
Effect of Fe_3_O_4_-siPD-L1@M_-BV2_ on polarization of microglia in orthotopic drug-resistant GBM tissue. **A** Distribution of microglia (marked with Iba-1, red color) in orthotopic drug-resistant GBM tissue. T: tumor tissue; N: normal tissue. **B** M1-type microglia (Iba-1 and CD16/32 co-positive cells) and M2-type microglia (Iba-1 and CD206 co-positive cells) in orthotopic drug-resistant GBM tissue. **C**, **D** Statistic analysis of M1 type and M2 type microglia in orthotopic drug-resistant GBM tissue. (n = 3, mean ± SD, ^*^P < 0.05, ^**^P < 0.01)

Finally, Fe_3_O_4_-siPD-L1@M_-BV2_ dose-dependently increased the contents of TNF-α, IFN-γ, IL-6 and IL-12 but decreased the contents of IL-10 in orthotopic drug-resistant GBM cells. Fe_3_O_4_-siPD-L1@M_-BV2_ + magnet significantly increased the contents of TNF-α, IFN-γ, IL-6 and IL-12 in comparison with Fe_3_O_4_-siPD-L1@M_-BV2_ did (Fig. 8I–M). Moreover, immunofluorescence staining showed that a large amount of microglia was mainly distributed in orthotopic drug-resistant GBM tissue (Fig. 9A). Fe_3_O_4_-siPD-L1, Fe_3_O_4_-siPD-L1@M_-BV2_ and Fe_3_O_4_-siPD-L1@M_-BV2_ + magnet significantly increased the number of Iba-1^+^CD16/32^+^ cells (M1 type microglia) in drug-resistant GBM tissue in comparison with normal saline and Fe_3_O_4_-siNC. The number of Iba-1^+^CD16/32^+^ cells was the highest in Fe_3_O_4_-siPD-L1@M_-BV2_ + magnet treatment group (Fig. 9B, C). Furthermore, as compared with normal saline and Fe_3_O_4_-siNC, Fe_3_O_4_-siPD-L1, Fe_3_O_4_-siPD-L1@M_-BV2_ and Fe_3_O_4_-siPD-L1@M_-BV2_ + magnet significantly reduced the number of Iba-1^+^CD206^+^ cells (M2 type microglia) in drug-resistant GBM tissue. The number of Iba-1^+^CD206^+^ cells was the lowest in Fe_3_O_4_-siPD-L1@M_-BV2_ + magnet treatment group (Fig. 9B, D).

### Preliminary safety evaluation of Fe_3_O_4_-siPD-L1@M_-BV2_ in orthotopic drug-resistant GBM mice

**Fig. 10:**
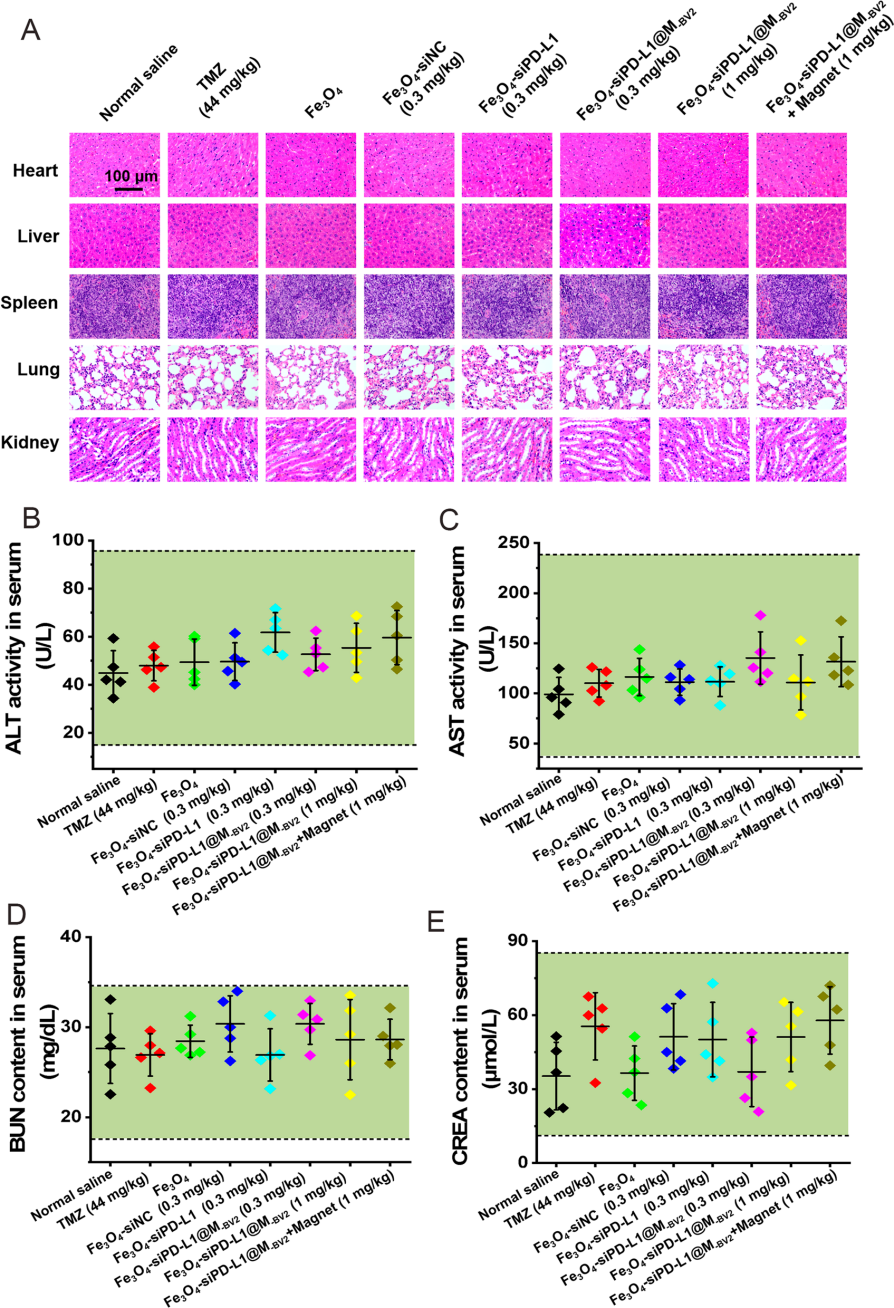
Safety evaluation of Fe_3_O_4_-siPD-L1@M_-BV2_ in orthotopic drug-resistant GBM mice. **A** *H&E* staining of heart, liver, spleen, lung and kidney tissue in orthotopic drug-resistant GBM mice. **B**, **C** Effects of Fe_3_O_4_-siPD-L1@M_-BV2_ on ALT and AST activity in serum of orthotopic drug-resistant GBM mice. **D**, **E** Effects of Fe_3_O_4_-siPD-L1@M_-BV2_ on content of BUN and CREA in serum of orthotopic drug-resistant GBM mice. The green area indicates the normal ranges. (n = 5, mean ± SD)

On the 24th day after plantation of Luc-GL261/TR cell, *H&E* staining showed that no obvious abnormal morphological changes were observed in the heart, liver, spleen, lung and kidney in all treatment groups (Fig. 10A). Biochemical analysis further showed that ALT and AST activities, BUN and CREA contents in mice serum in each treatment group were all within the normal range (Fig. 10B–E).

## Discussion

The PD-1/PD-L1 signaling pathway plays an important role in the tumor immune microenvironment [46]. The activation of PD-1/PD-L1 signaling pathway induces the apoptosis of the T_eff_ cell. Blocking the PD-1/PD-L1 signaling pathway activates T_eff_ cell, thus inhibiting tumor growth [47]. However, clinical data show that only less than 10% of patients with GBM response to immunotherapy because of the low immunogenicity of GBM [10, 48]. It has been shown that ferroptosis of cancer cells release high-mobility group box 1 (HMGB1) in an autophagy dependent manner [49], which increases the immunogenicity of cancer cells and promotes the maturation of DC cells [50–52]. The matured DC cells presents antigen to T lymphocytes and activates innate and adaptive immunity.

The key features of ferroptosis are the overload of Fe^2+^, depletion of GSH, the decrease of GPX4 activity and the consequent lipid peroxidation of cell membrane [53]. The results of MTT assay showed that Fe_3_O_4_-siPD-L1@M_-BV2_ decreased GL261/TR cell activity in a concentration-dependent manner. However, the inhibitory effect of Fe_3_O_4_-siPD-L1@M_-BV2_ on GL261/TR cell activity was significantly reduced in the present of ferroptosis inhibitors. After GL261/TR cells were incubated with Fe_3_O_4_-siPD-L1@M_-BV2_, the content of GSH and the protein expression of GPX4 in GL261/TR cells significantly decreased, which attenuated the ability of GL261/TR cells to clear intracellular ROS. This led to the increase of ROS level and lipid peroxidation in GL261/TR cells. However, in the present of ferroptosis inhibitor, the lipid peroxidation induced by Fe_3_O_4_-siPD-L1@M_-BV2_ in GL261/TR cells was significantly attenuated. Moreover, in vivo experimental results indicated that as compared with Fe_3_O_4_, the GSH content and protein expression of GPX4 and xCT were markedly reduced, and the ROS level and Fe^2+^ content was significantly increased in orthotopic drug-resistant GBM tissue in Fe_3_O_4_-siPD-L1@M_-BV2_ and Fe_3_O_4_-siPD-L1@M_-BV2_ + magnet treatment group. Those results demonstrated that ferroptosis was involved in the inhibitory effect of Fe_3_O_4_-siPD-L1@M_-BV2_ on GL261/TR cell activity. Fe_3_O_4_-siPD-L1@M_-BV2_ induced the ferroptosis of orthotopic drug-resistant GBM cells by increasing Fe^2+^ content and reducing the GPX4 protein expression.

When Fe_3_O_4_-siPD-L1@M_-BV2_ was co-cultured with GL261/TR cell, it significantly facilitated the maturation of DC cells. Ferroptosis inhibitors significantly inhibited the maturation of DC cells induced by Fe_3_O_4_-siPD-L1@M_-BV2_, suggesting that ferroptosis of GL261/TR cell induced by Fe_3_O_4_-siPD-L1@M_-BV2_ promoted the maturation of DC cells. Meanwhile, the in vitro experiment indicated that IFN-γ significantly enhanced ferroptosis of GL261/TR cells and the maturation of DC cells induced by Fe_3_O_4_-siPD-L1@M_-BV2_. Moreover, the in vivo experiment indicated that IFN-γ content and the number of matured DC cells in orthotopic drug-resistant GBM tissue significantly increased in Fe_3_O_4_-siPD-L1@M_-BV2_ group and Fe_3_O_4_-siPD-L1@M_-BV2_ + magnet group. Fe_3_O_4_-siPD-L1@M_-BV2_ reduced the protein expression of PD-L1 and increased the ratio between T_eff_ cell and T_reg_ cell in orthotopic drug-resistant GBM tissue. Those results suggested that T_eff_ cell was activated by Fe_3_O_4_-siPD-L1@M_-BV2_ through blocking PD-1/PD-L1 signal pathway, resulting in the release of IFN-γ. IFN-γ released from activated T_eff_ cell enhanced the ferroptosis of Fe_3_O_4_-siPD-L1@M_-BV2_, which promoted the maturation of DC cells in orthotopic drug-resistant GBM tissue. This activated the T_eff_ cell in turn and subsequently inhibited the growth of orthotopic drug-resistant GBM.

Clinical studies have shown that there is a large number of microglia in GBM tissue [54, 55]. GBM tissue promotes the polarization of microglia toward anti-inflammatory M2 type [11]. Studies have also shown that PD-L1 antibody activate mice macrophages to secrete more TNF-α and IL-12, which induce polarization of macrophages toward a pro-inflammatory M1 type [56, 57]. The in vivo experimental results showed that Fe_3_O_4_-siPD-L1@M_-BV2_ significantly increased the content of IFN-γ, TNF-α and IL-12 in orthotopic drug-resistant GBM tissue. The ratio between M1 type microglia and M2 type microglia in orthotopic drug-resistant GBM tissue was significantly increased by Fe_3_O_4_-siPD-L1@M_-BV2_. The above results demonstrated that Fe_3_O_4_-siPD-L1@M_-BV2_ polarized M2 type microglia to M1 type microglia through increasing IFN-γ, TNF-α and IL-12 content in orthotopic drug-resistant GBM tissue.

Improving the stability in blood circulation and targeting to GBM is the key point for siPDL-L1 to play its role in vivo. Pharmacokinetic results showed that Fe_3_O_4_-siPD-L1@M_-BV2_ markedly increased the stability of siPD-L1 in blood. In vivo bioluminescence imaging results indicated that the coating of microglia membrane improved the brain targeting of Fe_3_O_4_ nanoparticles. LSCM observation indicated that Fe_3_O_4_-siPD-L1@M_-BV2_ selectively distributed in the orthotopic drug-resistant GBM tissue. In addition, as compared with Fe_3_O_4_-siPD-L1, the intracellular siPD-L1 in orthotopic drug-resistant GBM tissue was significantly increased after Fe_3_O_4_-siPD-L1@M_-BV2_ was administrated. Meanwhile, the content of Fe^2+^ in orthotopic drug-resistant GBM tissue was also significantly increased by Fe_3_O_4_-siPD-L1@M_-BV2_. Those results demonstrated that Fe_3_O_4_-siPD-L1@M_-BV2_ exhibited good targeting for orthotopic drug-resistant GBM tissue. siPD-L1 and Fe^2+^ were simultaneously delivered to orthotopic drug-resistant GBM by Fe_3_O_4_-siPD-L1@M_-BV2_. Due to the paramagnetism of Fe_3_O_4_ nanoparticle, the brain targeting rate and the accumulation of Fe_3_O_4_-siPD-L1@M_-BV2_ in orthotopic drug-resistant GBM tissue was further increased under external magnetic field.

The in vivo imaging showed that Fe_3_O_4_-siPD-L1@M_-BV2_ was mainly distributed in brain and liver, while less Fe_3_O_4_-siPD-L1@M_-BV2_ was distributed in kidney and other tissue after intravenous administration. The amount of Fe_3_O_4_-siPD-L1@M_-BV2_ in brain tissue reached its maximum value at 12 h after administration. The distribution of Fe_3_O_4_-siPD-L1@M_-BV2_ in liver and brain significantly decreased at 24 h after administration. The above results indicated that Fe_3_O_4_-siPD-L1@M_-BV2_ and Fe_3_O_4_-siPD-L1@M_-BV2_ + megnet did not accumulate in the liver and kidney after a single tail vein injection. On the 24th day after plantation of Luc-GL261/TR cell, *H&E* staining and biochemical analysis indicated when Fe_3_O_4_-siPD-L1@M_-BV2_ and Fe_3_O_4_-siPD-L1@M_-BV2_ + megnet were intravenously injected into orthotopic drug-resistant GBM mice for 4 times within 12 days, they did not cause significant damage to heart, liver, spleen, lung and kidney in orthotopic drug-resistant GBM mice. However, this just was a preliminary safety evaluation. It is not certain yet if hepatorenal toxicity can be caused by Fe_3_O_4_-siPD-L1@M_-BV2_ and Fe_3_O_4_-siPD-L1@M_-BV2_ + megnet when its dose is greater than 1 mg/kg or more than 4 times of administration. This needs further study.

Western blot results showed that CX3CL1 and CSF-1 were highly expressed in GL261/TR cells, while CX3CR1 and CSF-1R were highly expressed in the membrane of microglia and surface of Fe_3_O_4_-siPD-L1 nanoparticles. In vitro studies showed that in the existence of chemokine CSF-1 and CX3CL1, the efficiency of Fe_3_O_4_-siPD-L1@M_-BV2_ penetration in vitro BBB was significantly improved, and the accumulation of Fe_3_O_4_-siPD-L1@M_-BV2_ in GL261/TR cells was also markedly increased. In contrast, CSF-1 antibody, CX3CL1 antibody, CSF-1R antibody and CX3CR1 antibody significantly reduced the efficiency of Fe_3_O_4_-siPD-L1@M_-BV2_ penetration in vitro BBB and its accumulation in GL261/TR cells. The results demonstrated that the interaction between chemokines (CSF-1 and CX3CL1) secreted by GL261/TR cells and receptors (CSF-1R and CX3CR1) on the microglia membrane promoted Fe_3_O_4_-siPD-L1@M_-BV2_ to penetrate BBB and accumulate in GL261/TR cells.

## Conclusion

Fe_3_O_4_-siPD-L1@M_-BV2_ was actively delivered to orthotopic drug-resistant GBM cell through the interaction between microglia membrane and GBM cell. Fe_3_O_4_-siPD-L1@M_-BV2_ activated T_eff_ cell by blocking the PD-1/PD-L1 signaling pathway. The activated T_eff_ cell released IFN-γ to promote ferroptosis of GBM cells and subsequently facilitated the maturation of DC cells in orthotopic drug-resistant GBM tissue. The matured DC cells presented GBM antigens to T lymphocytes to activate T_eff_ cell in turn. Meanwhile, M2 type microglia was polarized to M1 type microglia by IFN-γ secreted via T_eff_ cell in orthotopic drug-resistant GBM tissue. Finally, a virtuous cycle between ferroptosis and immunotherapy was formed and promoted each other by Fe_3_O_4_-siPD-L1@M_-BV2_, which exerted a synergistic effect on the treatment of orthotopic drug-resistant GBM.

## Supplementary Information

**Additional file 1: Figure S1.** Diagram of the applied magnetic field in orthotopic drug-resistant GBM mice brain after Fe_3_O_4_-siPD-L1@M_-BV2_ was injected into the tail vein. **Figure S2.** Characterization of Fe_3_O_4_-siPD-L1@M_-BV2_. (A) Element mapping analysis diagram of Fe_3_O_4_. (B) The zeta potential of Fe_3_O_4_, Fe_3_O_4_-siPD-L1, M_-BV2_ and Fe_3_O_4_-siPD-L1@M_-BV2_. (C) Stability of Fe_3_O_4_-siPD-L1@M_-BV2_ in water, PBS and 10% FBS solution. (D) TEM image of Fe_3_O_4_. (E) TEM image of Fe_3_O_4_-siPD-L1@M_-BV2_. (F) Hemolysis phenomenon of Fe_3_O_4_-siPD-L1@M_-BV2_. (G) Statistic analysis of hemolysis of Fe_3_O_4_-siPD-L1@M_-BV2_. (n = 3, mean ± SD). **Figure S3.** The density of GL261/TR cells, HT-22 cells, BV2 cells and RAW264.7 cells after the cells incubated at 37 ℃ for 24 h. (A) The density of GL261/TR cells, HT-22 cells, BV2 cells and RAW264.7 cells observed by optical microscope. (B) The density of GL261/TR cells, HT-22 cells, BV2 cells and RAW264.7 cells counted by using cell counters. (n = 3, mean ± SD, ns: no significant difference). **Figure S4.** Statistic analysis of Fe_3_O_4_-FAM@M_-BV2_ uptake by GL261/TR cells, HT-22 cells, BV2 cells and RAW264.7 cells detected by flow cytometer (n = 3, mean ± SD, ^*^P < 0.05, ^**^P < 0.01, ns: no significant difference). **Figure S5.** The uptake mechanism of Fe_3_O_4_-FAM@M_-BV2_ on GL261/TR cells detected by flow cytometer. (A) Effects of different inhibitors on the uptake of Fe_3_O_4_-FAM@M_-BV2_ by GL261/TR cells. (B) Statistic analysis of Fe_3_O_4_-FAM@M_-BV2_ uptake by GL261/TR cells (n = 3, mean ± SD, ^**^P < 0.01). **Figure S6.** The effect of Fe_3_O_4_-siPD-L1@M_-BV2_ on protein expression of PD-L1 in GL261 cells. (n = 3, mean ± SD, ^*^P < 0.05, ^**^P < 0.01). **Figure S7.** The effect of Fe_3_O_4_-siPD-L1@M_-BV2_ on the viability of GL261/TR cells. (A) The effect of Fe_3_O_4_-siPD-L1@M_-BV2_ on the death and living GL261/TR cells. (B) Statistic analysis of death and living GL261/TR cells. (n = 3, mean ± SD, ^*^P < 0.05, ^**^P < 0.01, ns: no significant difference). **Figure S8.** The effect of IFN-γ on the xCT protein expression in GL261/TR cells. (n = 3, mean ± SD, ^**^P < 0.01). **Figure S9.** The resistance values between transwell donor chamber and recipient chamber within 12 h after drug administration. (n = 3, mean ± SD, ns: no significant difference). **Figure S10.** Statistic analysis of Fe_3_O_4_-FAM@M_-BV2_ uptake by GL261/TR cells after Fe_3_O_4_-FAM@M_-BV2_ penetrated in vitro BBB detected by flow cytometer. (n = 3, mean ± SD, ^**^P < 0.01). **Figure S11.** The distribution of Fe_3_O_4_-siPD-L1@M_-BV2_ in organs of orthotopic drug-resistant GBM mice observed by in vivo bioluminescence imaging. **Figure S12.** The effect of Fe_3_O_4_-siPD-L1@M_-BV2_ on body weight of orthotopic drug-resistant GBM mice. (n = 10, mean ± SD). **Figure S13.** The expression of invasion-related proteins in orthotopic drug-resistant GBM tissue after orthotopic drug-resistant GBM mice was treated with Fe_3_O_4_-siPD-L1@M_-BV2_. (A) The expression of invasion-related proteins in orthotopic drug-resistant GBM tissue detected by western blot. (B) Semi-quantitative analysis of invasion-related proteins. (n = 3, mean ± SD, ^*^P < 0.05, ^**^P < 0.01). **Figure S14.** Effect on the protein expression of GPX4 and PD-L1 in orthotopic drug-resistant GBM tissue after orthotopic drug-resistant GBM mice was treated with Fe_3_O_4_-siPD-L1@M_-BV2_. (A) Semi-quantitative analysis of GPX4 protein expression in orthotopic drug-resistant GBM tissue detected by immunofluorescence staining. (B) Semi-quantitative analysis of PD-L1 protein expression in orthotopic drug-resistant GBM tissue. (n = 3, mean ± SD, ^*^P < 0.05, ^**^P < 0.01, ns: no significant difference). **Figure S15.** Typical flow cytometric graph of the matured DC cells. **Figure S16.** Typical flow cytometric graph of CD3^+^CD8^+^IFN-γ^+^ T cells. **Figure S17.** Typical flow cytometric graph of CD4^+^CD25^+^FoxP3^+^ T cells.

## Data Availability

All data generated or analyzed during this study are included in this published article and its supplementary information file.

## Abbreviations

ALT: Alanine aminotransferase
AST: Aspartate aminotransferase
BBB: Blood brain barrier
BODIPY-C11: Boron difluoride pyrrole fluorescent dyes-C11
BUN: Urea nitrogen
BSA: Bovine albumin
CD11c-APC: Clusters of differentiation 11 c-Allophycocyanin
CD25-FITC: Clusters of differentiation 25-fluorescein isothiocyanate
CD3-APC: Clusters of differentiation 3-Allophycocyanin
CD4-PE-Cy7: Clusters of differentiation4-Phycoerythrin-Sulfo-Cyanine7
CD44: Clusters of differentiation 44
CD8-FITC: Clusters of differentiation 8-fluorescein isothiocyanate
CD80-FITC: Clusters of differentiation 80-fluorescein
CD86-PE: Clusters of differentiation86-Phycoerythrin
CREA: Creatinine
CX3CL1: C-X3-C motif chemokine ligand 1
CX3CR1: C-X3-C motif chemokine receptor 1
DAPI: 4’,6-Diamidino-2-phenylindole dihydrochloride
DFO: Deferoxamine
DHE: Dihydroethidium
DMEM: Dulbecco's modified eagle medium
DMSO: Dimethyl sulfoxide
E-cadherin: E-Ca^2^^+^dependent cell adhesion molecules
FoxP3-PE: Forkhead box P3-Phycoerythrin
Fer-1: Ferrostatin-1
GL261/TR: TMZ-resistant glioma cells
GSH: Glutathione
GM-CSF: Granulocyte–macrophage colony stimulating factor
GPX4: Glutathione Peroxidase 4
HMGB1: Human high-mobility group box-1
Iba-1: Ionized calcium binding adaptor molecule 1
HR: Hemolysis rate
Iba-1: Ionized calcium binding adaptor molecule 1
IL-6/10/12: Interleukin-6/10/12
IFN-γ: Interferon-γ
IFN-γ-PE-Cy7: Interferon-γ-Phycoerythrin-Sulfo-Cyanine7
LPO: Lipid peroxidation
LSCM: Laser scanning confocal microscopy
M_-BV2_: Microglial cell membrane
MGMT: O^6^-methylguanine-DNA-methyltransferase
MMP-9: Matrix metalloproteinases-9
MTT: 3-(4,5-Dimethylthiazol-2-yl)-2,5-diphenyltetrazolium bromide
N-cadherin: N-Ca^2^^+^dependent cell adhesion molecules
PBS: Phosphate buffer saline
PDI: Polydispersity index
PD-L1: Programmed cell death ligand-1
ROS: Reactive oxygen species
RPMI1640: Roswell Park Memorial Institute 1640
SDS-PAGE: Sulfate–polyacrylamide gel electrophoresis
TEM: Transmission electron microscope
TGF-β: Transforming growth factor-β
TNF-α: Tumor necrosis factor-α
Tris: Trimethylol aminomethane
xCT: Cystine/glutamic acid reverse transporter
