# Supplementary material for: Biomimetic GBM-targeted drug delivery system boosting ferroptosis for immunotherapy of orthotopic drug-resistant GBM
Source: J Nanobiotechnology. 2022 Mar 27;20:161. doi: 10.1186/s12951-022-01360-6 (PMC8962245; doi:10.1186/s12951-022-01360-6)
Supplement: Supplementary file 1 — Additional file 1: Figure S1. Diagram of the applied magnetic field in orthotopic drug-resistant GBM mice brain after Fe3O4-siPD-L1@M-BV2 was injected into the tail vein. Figure S2. Characterization of Fe3O4-siPD-L1@M-BV2. (A) Element mapping analysis diagram of Fe3O4. (B) The zeta potential of Fe3O4, Fe3O4-siPD-L1, M-BV2 and Fe3O4-siPD-L1@M-BV2. (C) Stability of Fe3O4-siPD-L1@M-BV2 in water, PBS and 10% FBS solution. (D) TEM image of Fe3O4. (E) TEM image of Fe3O4-siPD-L1@M-BV2. (F) Hemolysis phenomenon of Fe3O4-siPD-L1@M-BV2. (G) Statistic analysis of hemolysis of Fe3O4-siPD-L1@M-BV2. (n = 3, mean ± SD). Figure S3. The density of GL261/TR cells, HT-22 cells, BV2 cells and RAW264.7 cells after the cells incubated at 37 ℃ for 24 h. (A) The density of GL261/TR cells, HT-22 cells, BV2 cells and RAW264.7 cells observed by optical microscope. (B) The density of GL261/TR cells, HT-22 cells, BV2 cells and RAW264.7 cells counted by using cell counters. (n = 3, mean ± SD, ns: no significant difference). Figure S4. Statistic analysis of Fe3O4-FAM@M-BV2 uptake by GL261/TR cells, HT-22 cells, BV2 cells and RAW264.7 cells detected by flow cytometer (n = 3, mean ± SD, *P < 0.05, **P < 0.01, ns: no significant difference). Figure S5. The uptake mechanism of Fe3O4-FAM@M-BV2 on GL261/TR cells detected by flow cytometer. (A) Effects of different inhibitors on the uptake of Fe3O4-FAM@M-BV2 by GL261/TR cells. (B) Statistic analysis of Fe3O4-FAM@M-BV2 uptake by GL261/TR cells (n = 3, mean ± SD, **P < 0.01). Figure S6. The effect of Fe3O4-siPD-L1@M-BV2 on protein expression of PD-L1 in GL261 cells. (n = 3, mean ± SD, *P < 0.05, **P < 0.01). Figure S7. The effect of Fe3O4-siPD-L1@M-BV2 on the viability of GL261/TR cells. (A) The effect of Fe3O4-siPD-L1@M-BV2 on the death and living GL261/TR cells. (B) Statistic analysis of death and living GL261/TR cells. (n = 3, mean ± SD, *P < 0.05, **P < 0.01, ns: no significant difference). Figure S8. The effect of IFN-γ on the xCT protein expression [file 12951_2022_1360_MOESM1_ESM.docx]

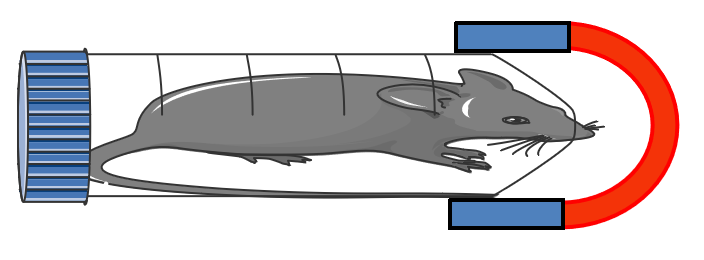


**Figure S1.** Diagram of the applied magnetic field in orthotopic drug-resistant GBM mice brain after Fe₃O₄-siPD-L1@M_-BV2_ was injected into the tail vein.


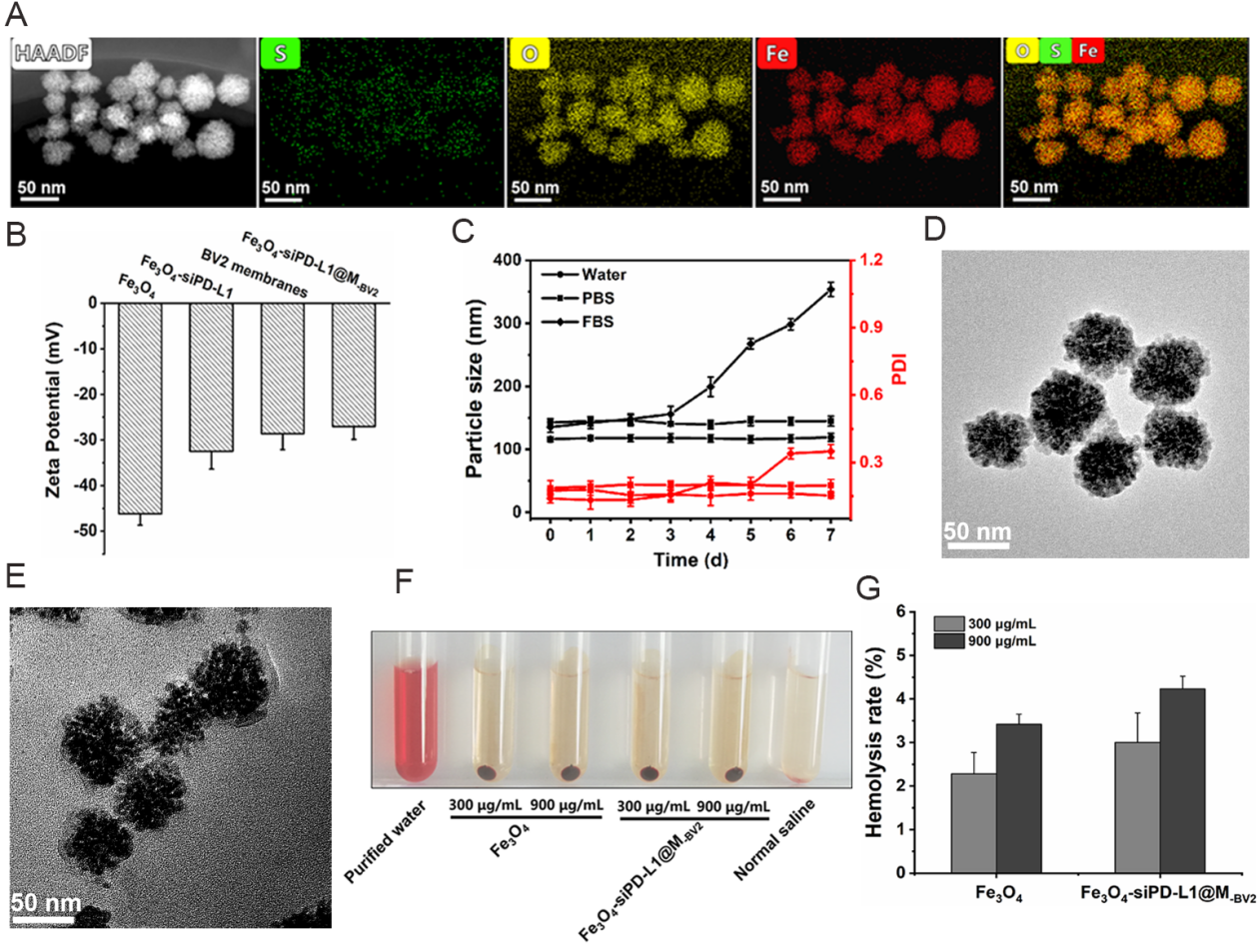


**Figure S2.** Characterization of Fe_3_O_4_-siPD-L1@M_-BV2_. (A) Element mapping analysis diagram of Fe_3_O_4_. (B) The zeta potential of Fe_3_O_4_, Fe_3_O_4_-siPD-L1, M_-BV2_ and Fe_3_O_4_-siPD-L1@M_-BV2_. (C) Stability of Fe_3_O_4_-siPD-L1@M_-BV2_ in water, PBS and 10% FBS solution. (D) TEM image of Fe_3_O_4_. (E) TEM image of Fe_3_O_4_-siPD-L1@M_-BV2_. (F) Hemolysis phenomenon of Fe_3_O_4_-siPD-L1@M_-BV2_. (G) Statistic analysis of hemolysis of Fe_3_O_4_-siPD-L1@M_-BV2_. (n=3, mean±SD).


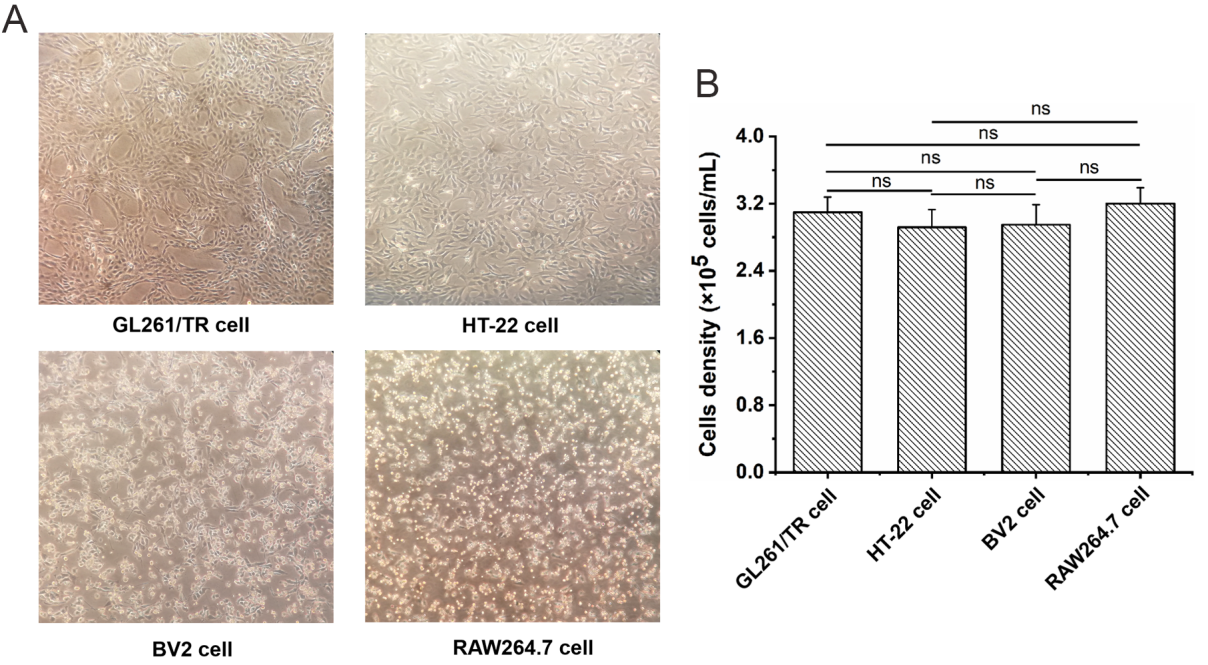


**Figure S3.** The density of GL261/TR cells, HT-22 cells, BV2 cells and RAW264.7 cells after the cells incubated at 37 ℃ for 24 h. (A) The density of GL261/TR cells, HT-22 cells, BV2 cells and RAW264.7 cells observed by optical microscope. (B) The density of GL261/TR cells, HT-22 cells, BV2 cells and RAW264.7 cells counted by using cell counters. (n=3, mean±SD, ns: no significant difference)


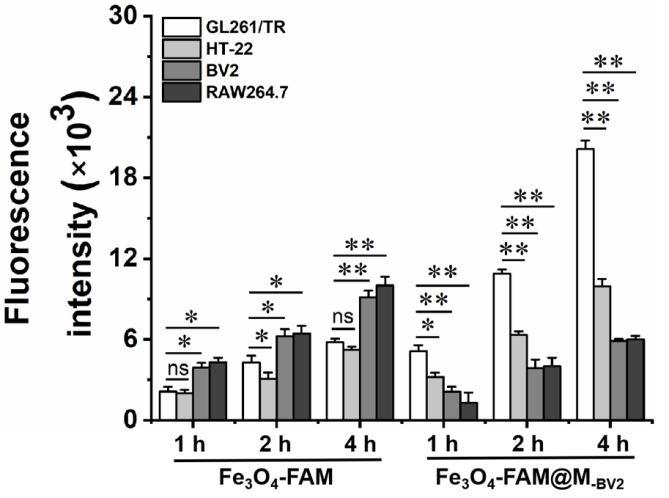


**Figure S4.** Statistic analysis of Fe_3_O_4_-FAM@M_-BV2_ uptake by GL261/TR cells, HT-22 cells, BV2 cells and RAW264.7 cells detected by flow cytometer (n=3, mean±SD, ^*^*P*＜0.05, ^**^*P*＜0.01, ns: no significant difference).


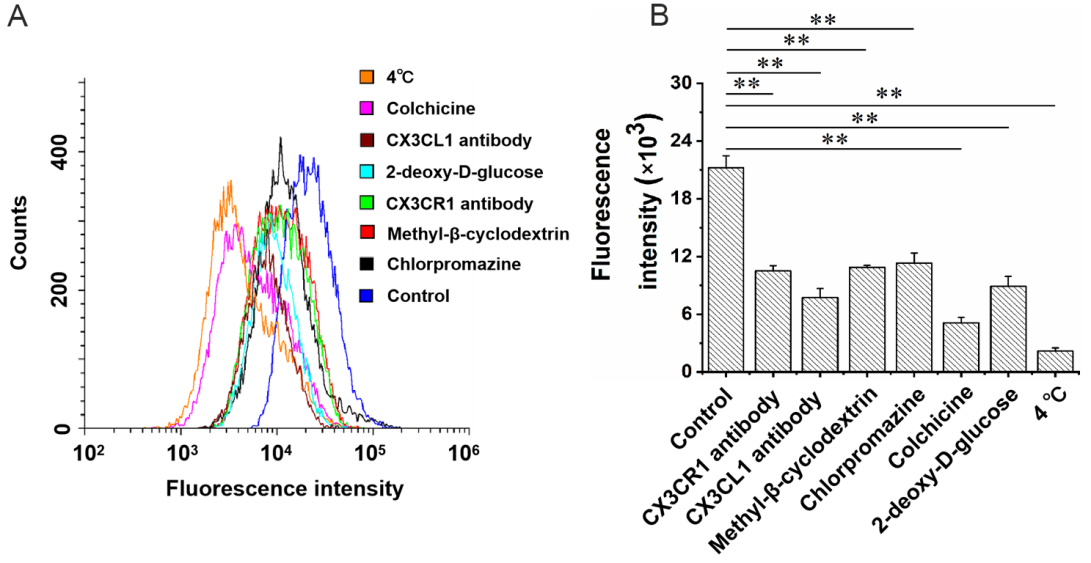


**Figure S5.** The uptake mechanism of Fe_3_O_4_-FAM@M_-BV2_ on GL261/TR cells detected by flow cytometer. (A) Effects of different inhibitors on the uptake of Fe_3_O_4_-FAM@M_-BV2_ by GL261/TR cells. (B) Statistic analysis of Fe_3_O_4_-FAM@M_-BV2_ uptake by GL261/TR cells (n=3, mean±SD, ^**^*P*＜0.01).


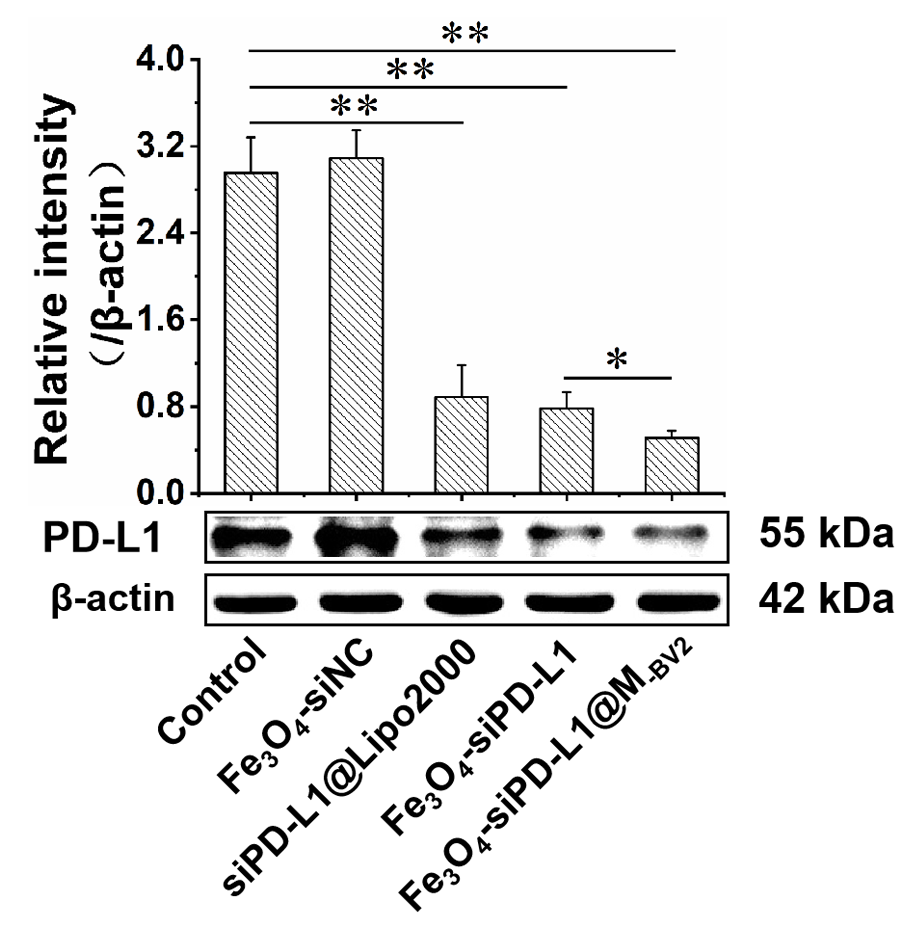


**Figure S6.** The effect of Fe_3_O_4_-siPD-L1@M_-BV2_ on protein expression of PD-L1 in GL261 cells. (n=3, mean±SD, ^*^*P*＜0.05, ^**^*P*＜0.01)


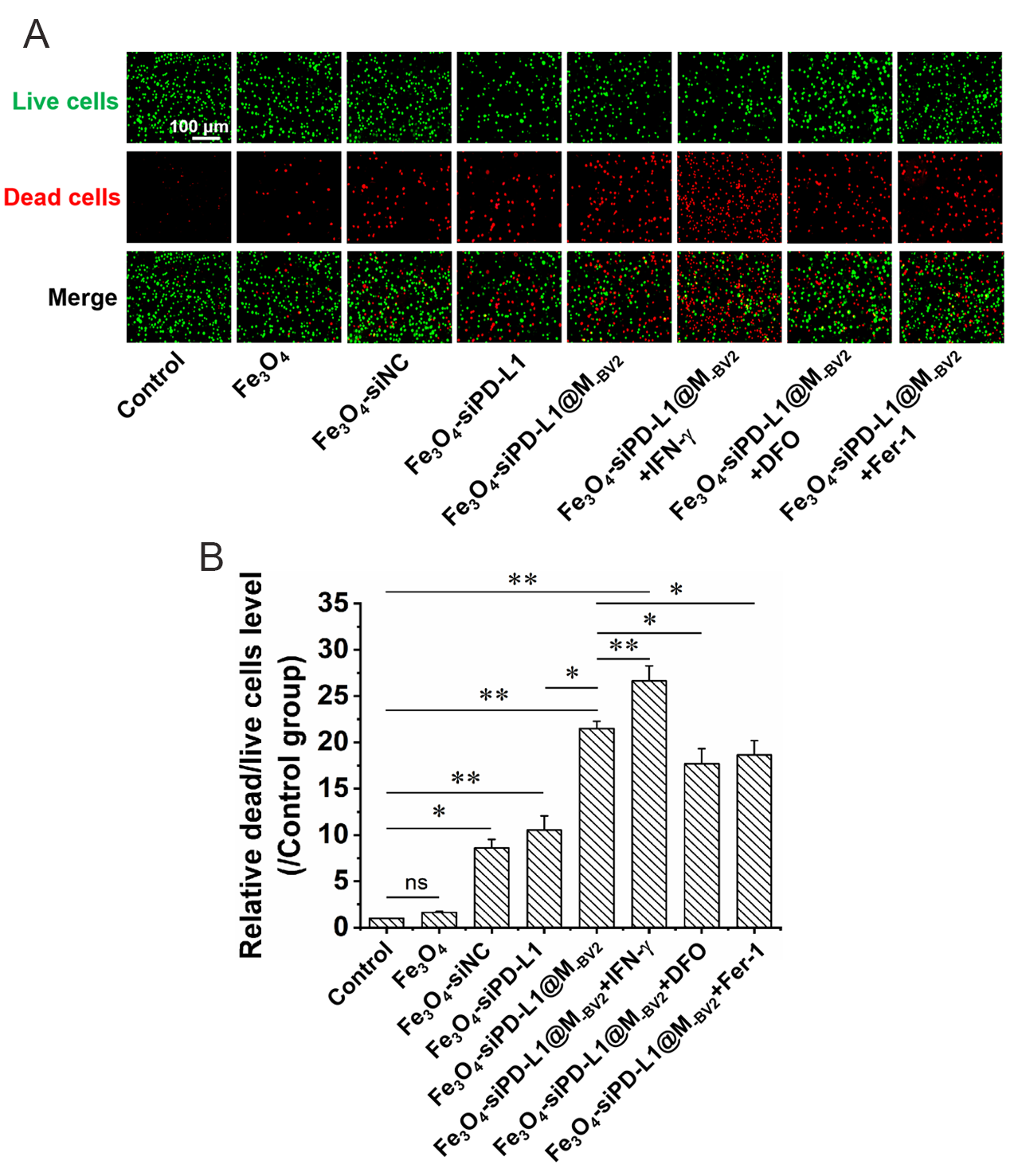


**Figure S7.** The effect of Fe_3_O_4_-siPD-L1@M_-BV2_ on the viability of GL261/TR cells. (A) The effect of Fe_3_O_4_-siPD-L1@M_-BV2_ on the death and living GL261/TR cells. (B) Statistic analysis of death and living GL261/TR cells. (n=3, mean±SD, ^*^*P*＜0.05, ^**^*P*＜0.01，ns: no significant difference).


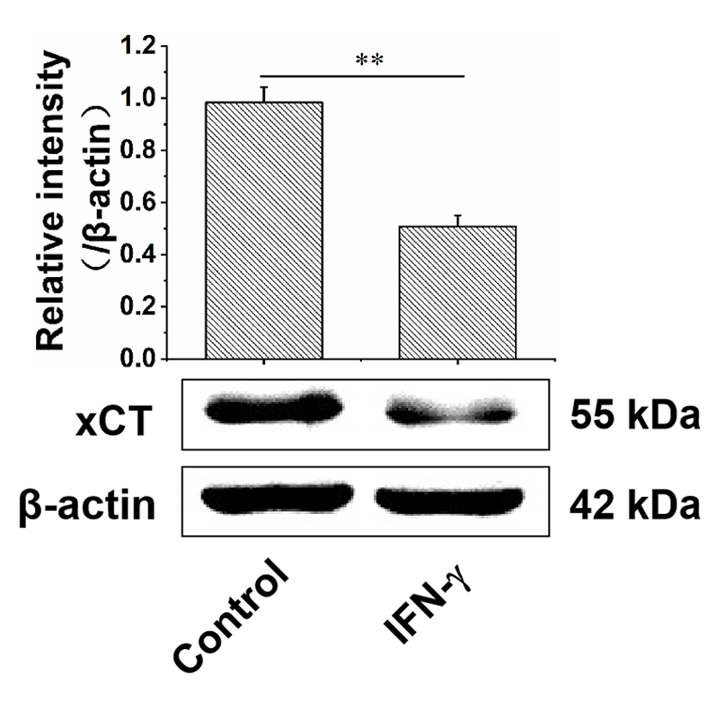


**Figure S8.** The effect of IFN-γ on the xCT protein expression in GL261/TR cells. (n=3, mean±SD, ^**^*P*＜0.01)


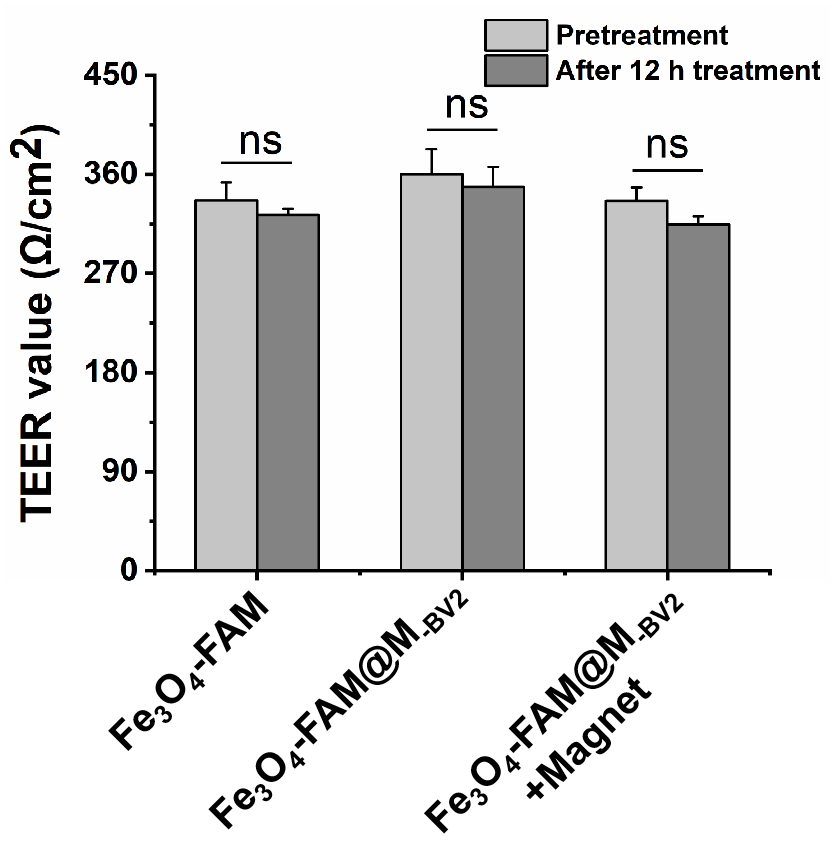


**Figure S9.** The resistance values between transwell donor chamber and recipient chamber within 12 h after drug administration. (n=3, mean±SD，ns: no significant difference)


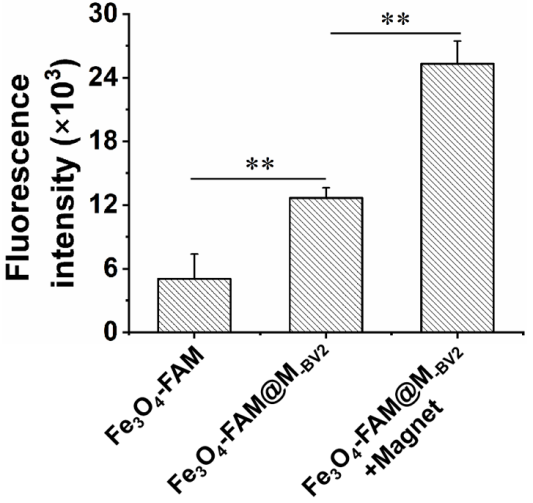


**Figure S10.** Statistic analysis of Fe_3_O_4_-FAM@M_-BV2_ uptake by GL261/TR cells after Fe_3_O_4_-FAM@M_-BV2_ penetrated *in vitro* BBB detected by flow cytometer. (n=3, mean±SD, ^**^*P*＜0.01).


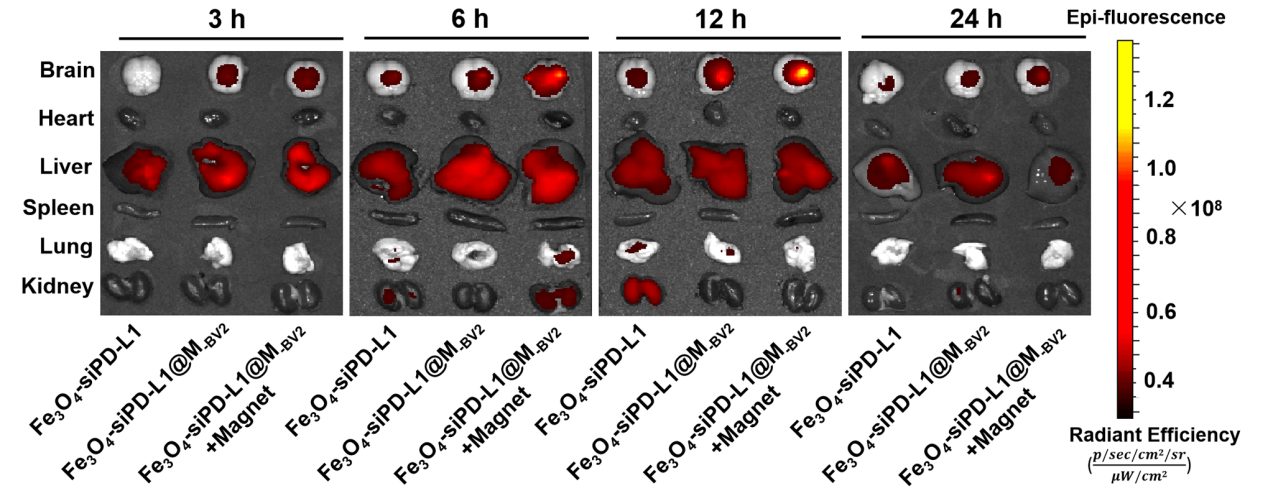


**Figure S11.**  The distribution of Fe_3_O_4_-siPD-L1@M_-BV2_ in organs of orthotopic drug-resistant GBM mice observed by *in vivo* bioluminescence imaging.





**Figure S12.** The effect of Fe_3_O_4_-siPD-L1@M_-BV2_ on body weight of orthotopic drug-resistant GBM mice. (n=10，mean±SD).


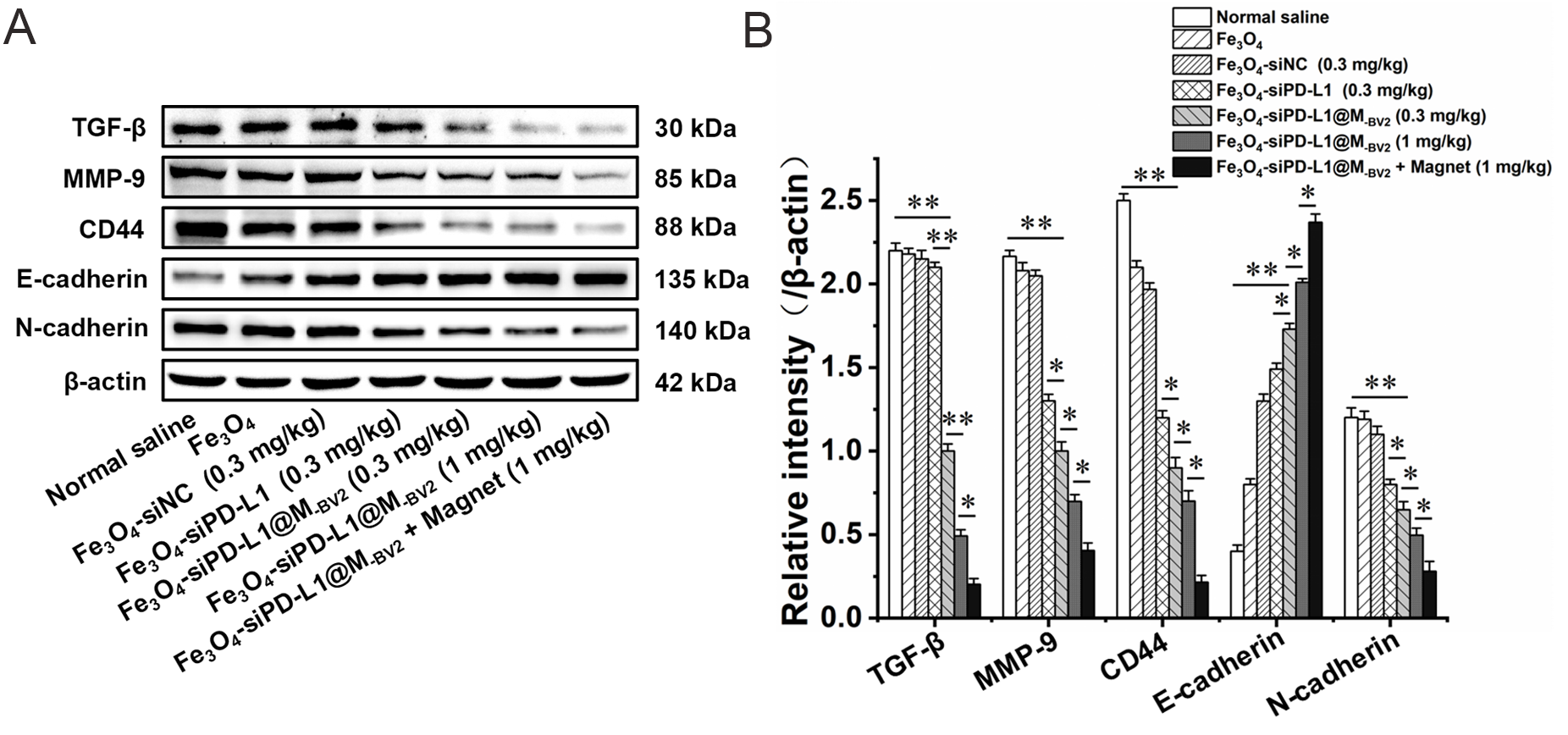


**Figure S13.** The expression of invasion-related proteins in orthotopic drug-resistant GBM tissue after orthotopic drug-resistant GBM mice was treated with Fe_3_O_4_-siPD-L1@M_-BV2_. (A) The expression of invasion-related proteins in orthotopic drug-resistant GBM tissue detected by western blot. (B) Semi-quantitative analysis of invasion-related proteins. (n=3，mean±SD, ^*^*P*＜0.05, ^**^*P*＜0.01).


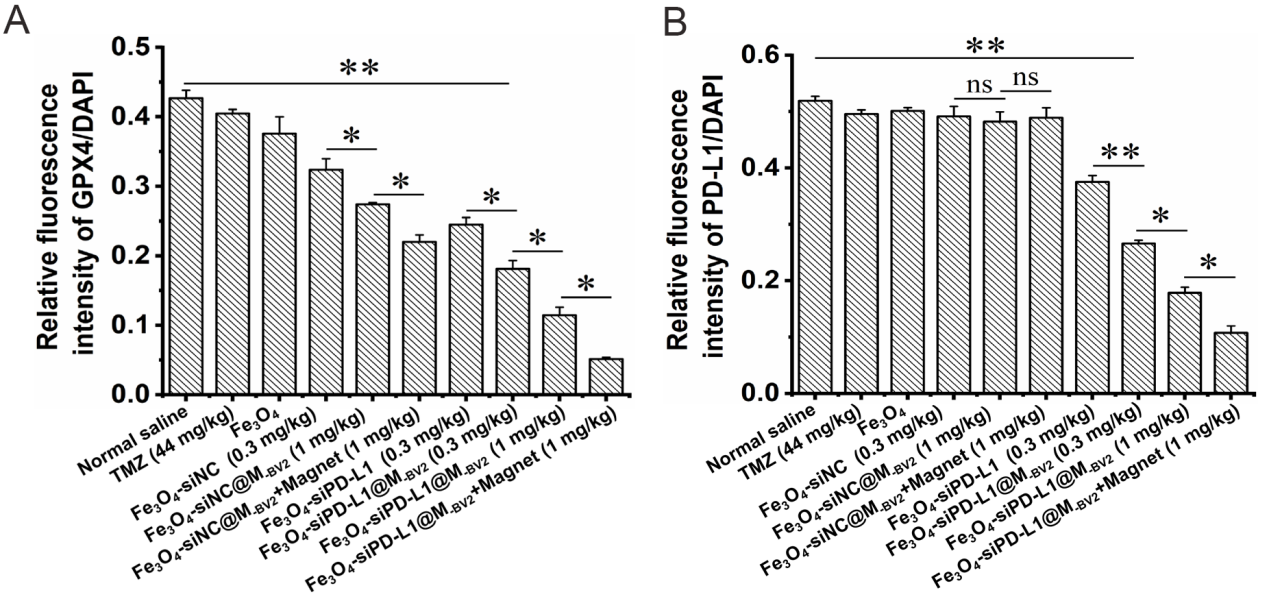


**Figure S14.** Effect on the protein expression of GPX4 and PD-L1 in orthotopic drug-resistant GBM tissue after orthotopic drug-resistant GBM mice was treated with Fe_3_O_4_-siPD-L1@M_-BV2_. (A) Semi-quantitative analysis of GPX4 protein expression in orthotopic drug-resistant GBM tissue detected by immunofluorescence staining. (B) Semi-quantitative analysis of PD-L1 protein expression in orthotopic drug-resistant GBM tissue. (n=3，mean±SD, ^*^*P*＜0.05, ^**^*P*＜0.01, ns: no significant difference).


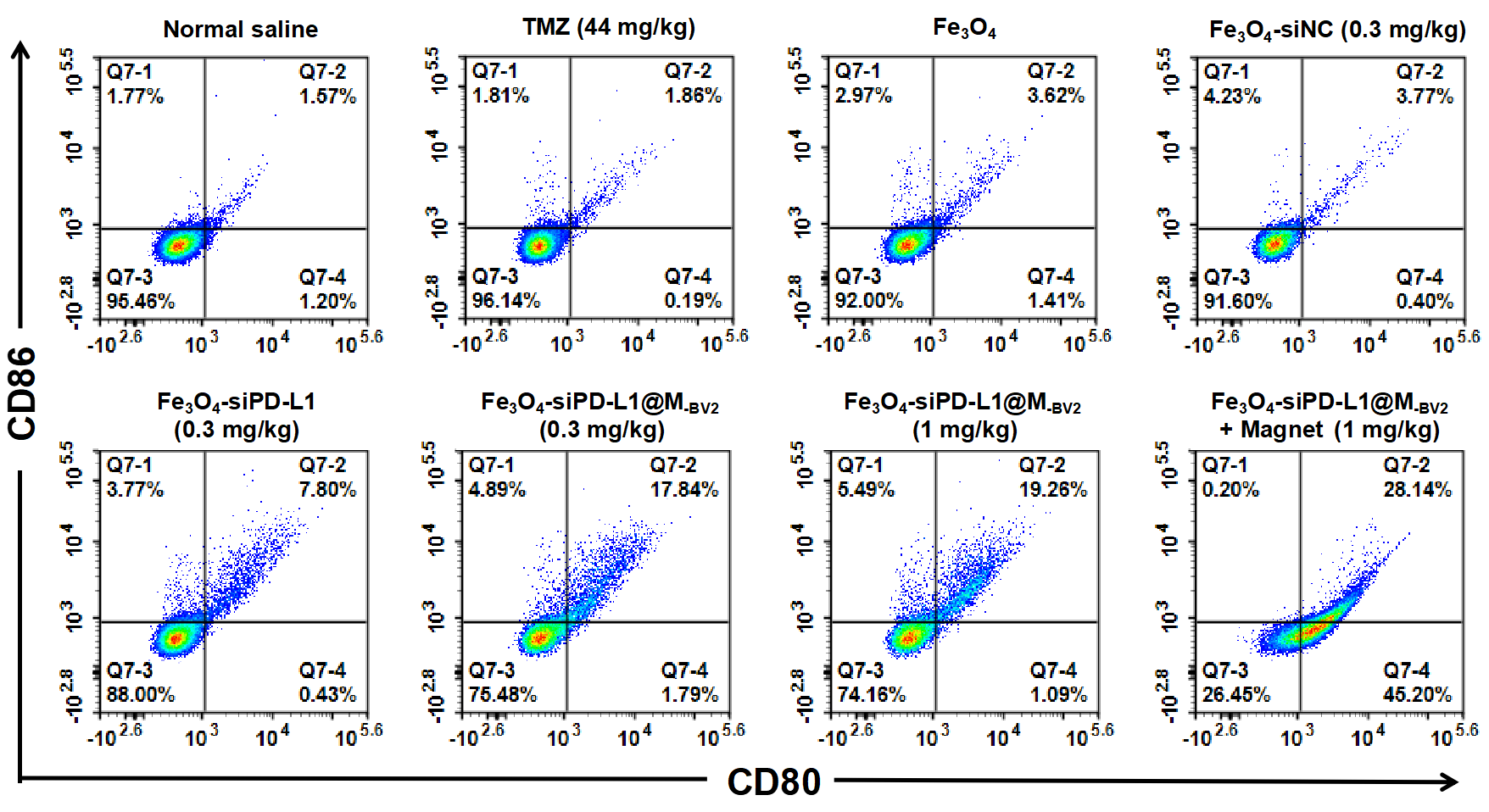


**Figure S15.** Typical flow cytometric graph of the matured DC cells.
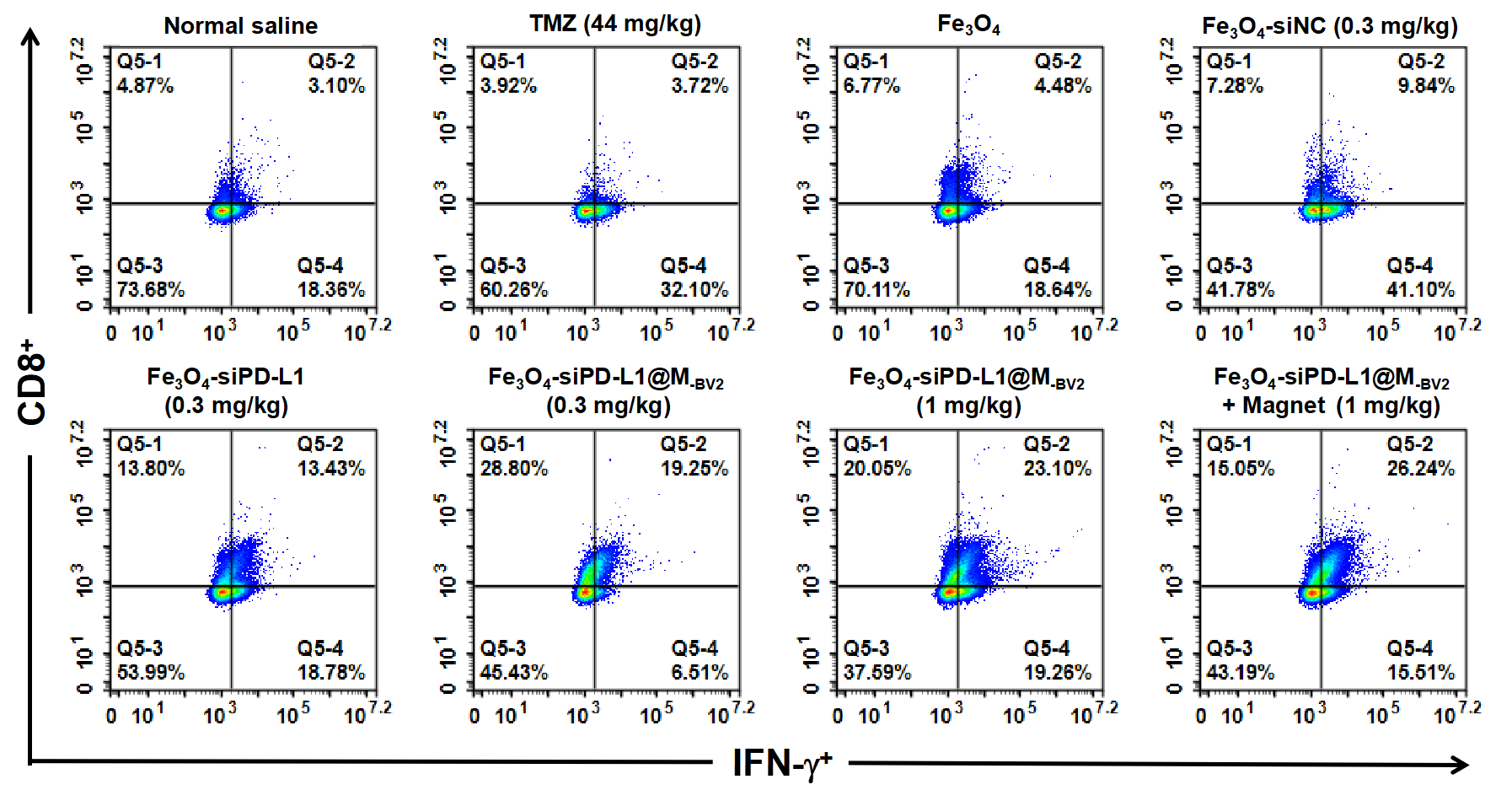


**Figure S16.** Typical flow cytometric graph of CD3^+^CD8^+^IFN-γ^+^ T cells.

**
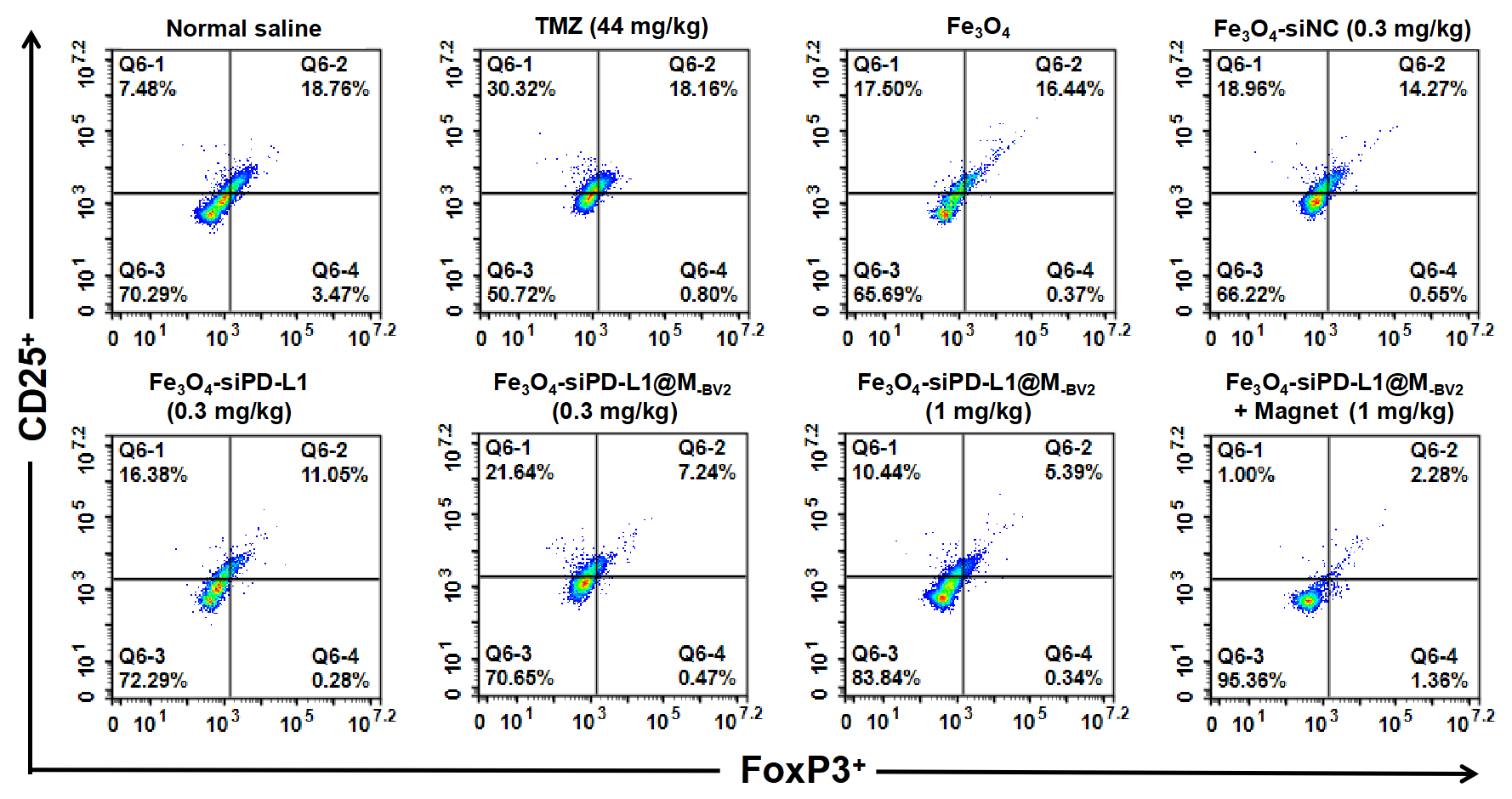
**

**Figure S17.** Typical flow cytometric graph of CD4^+^CD25^+^FoxP3^+^ T cells.
